# Protein Deimination Signatures in Plasma and Plasma-EVs and Protein Deimination in the Brain Vasculature in a Rat Model of Pre-Motor Parkinson’s Disease

**DOI:** 10.3390/ijms21082743

**Published:** 2020-04-15

**Authors:** Marco Sancandi, Pinar Uysal-Onganer, Igor Kraev, Audrey Mercer, Sigrun Lange

**Affiliations:** 1 Department of Pharmacology, UCL School of Pharmacy, London WC1N 1AX, UK; marco.sancandi.16@ucl.ac.uk (M.S.); a.mercer@ucl.ac.uk (A.M.); 2 Cancer Research Group, School of Life Sciences, University of Westminster, London W1W 6XH, UK; P.onganer@westminster.ac.uk; 3 Electron Microscopy Suite, Faculty of Science, Technology, Engineering and Mathematics, Open University, Milton Keynes MK7 6AA, UK; igor.kraev@open.ac.uk; 4 Tissue Architecture and Regeneration Research Group, School of Life Sciences, University of Westminster, London W1W 6XH, UK

**Keywords:** Peptidylarginine deiminases (PADs), protein deimination, extracellular vesicles (EVs), pre-motor Parkinson’s disease, microRNA (miR21, miR155, miR210)

## Abstract

The identification of biomarkers for early diagnosis of Parkinson’s disease (PD) is of pivotal importance for improving approaches for clinical intervention. The use of translatable animal models of pre-motor PD therefore offers optimal opportunities for novel biomarker discovery in vivo. Peptidylarginine deiminases (PADs) are a family of calcium-activated enzymes that contribute to protein misfolding through post-translational deimination of arginine to citrulline. Furthermore, PADs are an active regulator of extracellular vesicle (EV) release. Both protein deimination and extracellular vesicles (EVs) are gaining increased attention in relation to neurodegenerative diseases, including in PD, while roles in pre-motor PD have yet to be investigated. The current study aimed at identifying protein candidates of deimination in plasma and plasma-EVs in a rat model of pre-motor PD, to assess putative contributions of such post-translational changes in the early stages of disease. EV-cargo was further assessed for deiminated proteins as well as three key micro-RNAs known to contribute to inflammation and hypoxia (miR21, miR155, and miR210) and also associated with PD. Overall, there was a significant increase in circulating plasma EVs in the PD model compared with sham animals and inflammatory and hypoxia related microRNAs were significantly increased in plasma-EVs of the pre-motor PD model. A significantly higher number of protein candidates were deiminated in the pre-motor PD model plasma and plasma-EVs, compared with those in the sham animals. KEGG (Kyoto encyclopedia of genes and genomes) pathways identified for deiminated proteins in the pre-motor PD model were linked to “Alzheimer’s disease”, “PD”, “Huntington’s disease”, “prion diseases”, as well as for “oxidative phosphorylation”, “thermogenesis”, “metabolic pathways”, “*Staphylococcus aureus* infection”, gap junction, “platelet activation”, “apelin signalling”, “retrograde endocannabinoid signalling”, “systemic lupus erythematosus”, and “non-alcoholic fatty liver disease”. Furthermore, PD brains showed significantly increased staining for total deiminated proteins in the brain vasculature in cortex and hippocampus, as well as increased immunodetection of deiminated histone H3 in dentate gyrus and cortex. Our findings identify EVs and post-translational protein deimination as novel biomarkers in early pre-motor stages of PD.

## 1. Introduction

Identification of biomarkers for early diagnosis of Parkinson’s disease (PD) is essential for developing early clinical intervention strategies. The use of animal models that present symptoms comparable to those observed in PD patients in the early stages of the disease, prior to the appearance of motor dysfunctions, therefore offers promising avenues for novel biomarker discovery in vivo. Peptidylarginine deiminases (PADs) are a family of calcium-activated enzymes that contribute to protein misfolding, as well as changes in protein function, through post-translational deimination of arginine to citrulline [1,2]. Furthermore, PADs are an active regulator of extracellular vesicle (EVs) release [3,4,5,6,7]. Both protein deimination and EVs are gaining increasing attention in relation to neurodegenerative diseases [8,9], including in PD, while their roles in pre-motor PD have yet to be investigated. Increased PAD-mediated protein deimination is indeed observed in several autoimmune, chronic and neurodegenerative diseases, including in PD [8,9,10,11,12,13,14]. In PD, deiminated proteins have been detected in substantia nigra of post-mortem human brain samples [10]. Furthermore, in PD iPSC neuronal models, derived from fibroblasts of patients carrying α-synuclein triplication [15], an increase and change in deiminated protein patterns has also been reported [9].

EVs are a recognized contributor to neurodegenerative diseases, and both neurotoxic and neuroprotective roles via distribution of EV-mediated cargo, including microRNAs and misfolded proteins, have been implicated [16,17]. In addition, EVs may be usable as a “liquid biopsy” for identification of disease related biomarkers [8,16,17,18,19,20]. EVs have indeed recently been suggested as putative biomarkers in PD [21]. For example, PD patients have been shown to have greater amounts of circulating small EVs [22]. However, less is known about EVs in early and pre-motor stages of PD.

The current study aimed at profiling EVs and identifying protein candidates of deimination in plasma and plasma-EVs in a rat model of pre-motor PD to determine putative contributions of such post-translationally mediated changes in early stages of PD, including further downstream effects that may be related to disease progression. To this end, we used a toxin-induced pre-motor PD model that was shown to display non-motor symptoms in the absence of motor symptoms and pathologies mimicking those observed in patients (See 4.1 in the Methods section). EV-cargo was assessed for deiminated proteins as well as for three key microRNAs known to contribute to inflammation and hypoxia (miR21, miR155, and miR210), all of which have also been associated to neurodegenerative diseases and PD [23,24,25], but have not been related to, or assessed in, pre-motor PD before the current study. Epigenetic mechanisms of PD have indeed received increasing attention, including histone modifications, DNA methylation, as well as microRNA involvement [26,27]. Our findings identify EVs and protein deimination as novel markers in pre-motor stages of PD.

## 2. Results

### 2.1. Circulating Plasma Extracellular Vesicles (EVs) are Significantly Increased in the Pre-Motor Parkinson’s Disease (PD) Model Compared with Sham-Treated Rats

**Figure 1:**
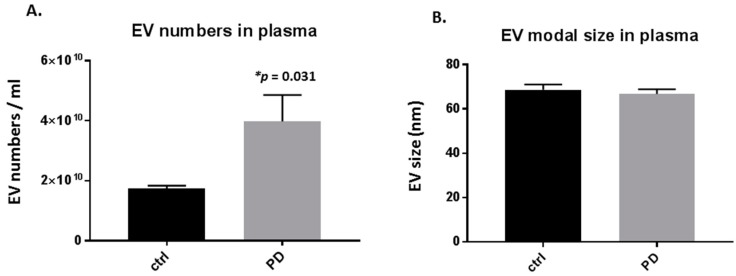
Plasma-extracellular vesicles (EVs) are elevated in pre-motor Parkinson’s disease (PD) model rats. (**A**) The number of circulating plasma-EVs was significantly increased in plasma of the pre-motor PD model rats, compared with that in plasma of sham control rats (*p* < 0.05; unpaired t-test). (**B**) Modal size of plasma-EVs did not differ between plasma from the pre-motor PD rat model compared to control shams. Exact *p*-values are indicated (*n* = 3 biological replicates for all; ctrl = sham; PD = pre-motor PD models).

Significant changes were observed in the number of circulating plasma-EVs in the pre-motor PD, compared with sham-treated rats, with a 2-fold increase in plasma-EVs (*p* = 0.031; Figure 1A). Modal plasma-EV size did not show a significant change between the pre-motor PD and shams (Figure 1B).

**Figure 2:**
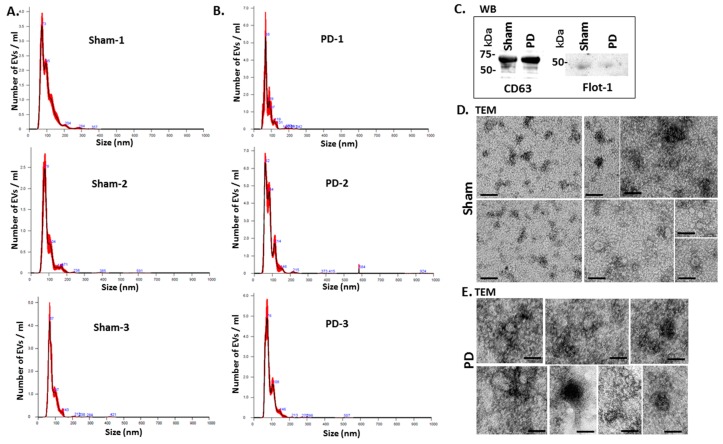
EV characterisation from rat plasma. (**A**) Representative Nanosight graphs showing nanoparticle tracking analysis (NTA) analysis of plasma EV profiles from sham-treated rats (Sham; *n* = 3). (**B**) Representative Nanosight graphs showing NTA analysis of plasma EV profiles from the pre-motor PD rat models (PD; *n* = 3). (**C**) Western blotting (WB) confirms that rat plasma-EVs are positive for the EV-specific markers CD63 and flotillin-1 (Flot-1); the molecular weight standard is indicated in kilo Daltons (kDa). (**D**,**E**) Transmission electron microscopy (TEM) images showing EVs isolated from sham (**D**) and pre-motor PD model (PD; (**E**)) rat plasma, revealing typical EV morphology; composite images are shown and the scale bar represents 50 nm in all images. In the NTA curves the black line represents the mean of the 5 repetitive readings per individual sample and the red line represents standard error (+/−) between those same 5 readings per sample. Each treatment group was measured in 3 biological replicates (sham; PD = pre-motor PD models).

Figure 2 shows representative nanoparticle tracking analysis (NTA) profiles of EV size distribution from shams and pre-motor PD animals (Figure 2A,B). Additional EV characterisation was carried out by western blotting (WB) using the EV-specific markers CD63 and flotillin-1 (Flot-1), which showed positive for the rat EVs (Figure 2C), as well as by transmission electron microscopy (TEM), revealing typical EV morphology (Figure 2D,E).

### 2.2. Inflammatory and Hypoxia Related microRNA EV-Cargo is Increased in Plasma of Pre-Motor PD Models

**Figure 3:**
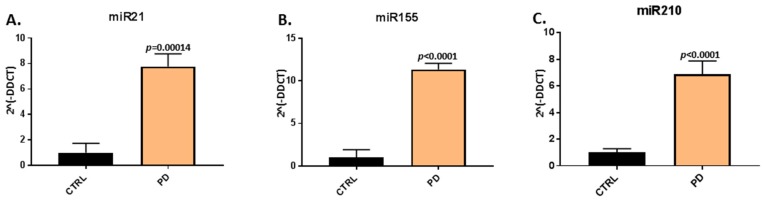
MicroRNA analysis of inflammatory and hypoxia associated microRNAs in circulating plasma-EVs of pre-motor PD and sham animals. (**A**) Significantly increased relative expression of the inflammatory miR21 was observed in plasma-EVs of the pre-motor PD rats, compared with shams. (**B**) Significantly increased relative expression of the inflammatory miR155 was observed in plasma-EVs of pre-motor PD rats, compared with shams. (**C**) The relative expression of the hypoxia related miR210 was significantly increased in plasma-EVs of pre-motor PD rats, compared with those of sham-treated animals. Results are represented as relative microRNA (miR) expression compared to the internal control miRs (2^Λ^(-DDCT)); exact *p*-values are indicated, error bars show SD (*n* = 3 biological and three technical replicates for all; ctrl = sham; PD = pre-motor PD models).

When assessing EV-cargo for two inflammatory (miR21, miR155) and one hypoxia related microRNA (miR210), a significant increase in relative expression was found for all three microRNAs in plasma-EVs of the pre-motor PD models, compared with that in sham-treated animals (Figure 3). The pro-inflammatory miR21 was increased by 7.77-fold (*p* = 0.00014) in pre-motor PD plasma-EVs, compared with shams (Figure 3A); the pro-inflammatory miR155 was 11.34-fold increased (*p* < 0.0001) in the pre-motor PD plasma-EVs, compared with shams (Figure 3B); and the hypoxia-related miR210 was 6.88-fold increased (*p* < 0.0001) in the pre-motor PD plasma-EVs, compared with shams (Figure 3C).

### 2.3. Deiminated Protein Targets and Kyoto Encyclopedia of Genes and Genomes (KEGG) Pathways Enriched in Deiminated Proteins Differ between Pre-Motor PD and Sham-Treated Rat Plasma and Plasma-EVs

**Figure 4:**
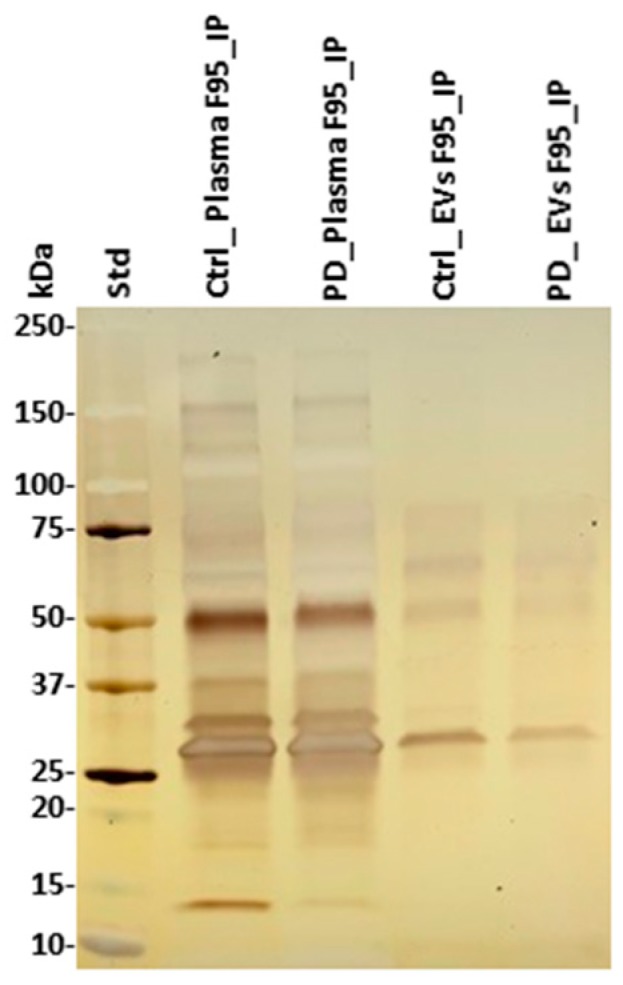
F95-enriched fractions from plasma and plasma-EVs from pre-motor PD model and sham rats. Silver stained sodium dodecyl sulfate polyacrylamide gel electrophoresis (SDS-PAGE) gel (4–20% gradient gel), showing F95-enriched protein bands from whole plasma and plasma-EVs in pre-motor PD and sham (control) rats. The protein standard is indicated on the far left in kilodaltons (kDa) (ctrl = sham; PD = pre-motor PD models).

Deiminated proteins isolated by F95-enrichment were assessed by silver staining, revealing a number of protein bands in the range of 15–200 kDa (Figure 4). There was no obvious difference observed in protein banding patterns of F95-enriched protein eluates (F95_IP) between the sham (ctrl) and pre-motor PD-model; neither in plasma nor plasma-EVs, while concentration of F95-enriched proteins unexpectedly seemed slightly higher in the sham (ctrl) animals, without verifying specific target proteins of deimination at this point. The F95-enriched eluates were further assessed by liquid chromatography mass spectrometry (LC-MS/MS) analysis for identification of differences in deiminated target proteins in sham, compared with pre-motor PD animals, both for plasma and plasma-EVs.

**Figure 5:**
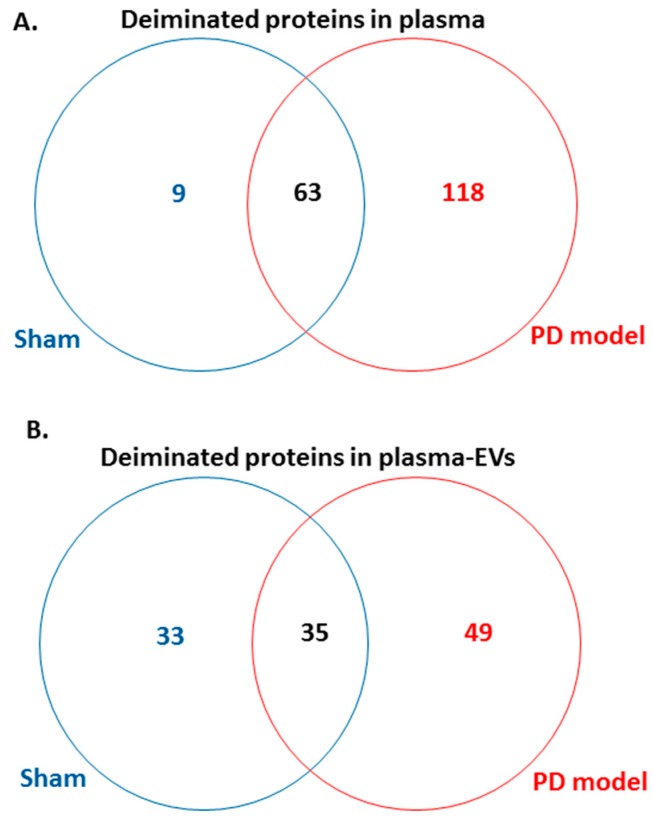
Venn diagram showing deiminated protein hits identified in plasma and plasma-EVs of pre-motor PD model and of sham rats. (**A**) A considerable higher number of deiminated protein hits was identified in plasma of the pre-motor PD model rats, compared with that in shams, with some overlapping protein hits. (**B**) A higher number of protein hits was identified to be deiminated in circulating plasma-EVs of the pre-motor PD rat models, compared with that in shams, with some hits overlapping.

Following LC-MS/MS analysis, F95-enriched protein candidates identified were analysed for protein–protein interaction networks using STRING (https://string-db.org/). STRING analysis revealed some differences in Kyoto encyclopedia of genes and genomes (KEGG) pathways enriched in deiminated proteins between the pre-motor PD model and the shams, both for plasma-EVs as well as whole plasma. Overall, more protein hits were identified as deiminated in the pre-motor PD models, both in EVs and whole plasma, compared with shams, with some common targets but some specific to either shams or the pre-motor PD models as summarised in the Venn diagram in Figure 5. In summary, 118 protein hits were specific to the pre-motor PD model whole plasma and 9 deiminated protein hits were specific to sham plasma (Figure 5A; Table 1 and Table S1). In plasma-EVs of the pre-motor PD rats, 49 deiminated protein hits were specific, while 33 deimination hits were specific to plasma-EVs from shams (Figure 5B, Table 2, and Table S2).

**Figure 6:**
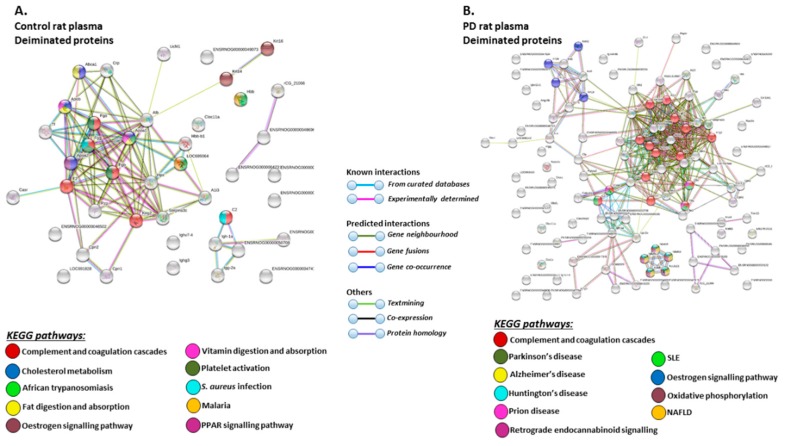
Kyoto encyclopedia of genes and genomes (KEGG) pathways for deiminated proteins identified in plasma of sham and pre-motor PD model rats. (**A**) KEGG pathways identified in circulating EVs of sham-treated (control) rat plasma are highlighted. Colour codes for nodes included in the figure. (**B**) KEGG pathways identified for deiminated proteins in circulating EVs of pre-motor PD model rat plasma are highlighted; notably pathways for PD and other neurodegenerative disease are identified for deiminated proteins in the pre-motor PD plasma-EVs. See colour codes for nodes and for connecting lines in the figure.

STRING analysis of the KEGG pathways identified for deiminated protein hits in total plasma of the pre-motor PD model and sham-treated rat plasma respectively, is shown in Figure 6 (Figure 6A,B; see also Table 1 and Table 2 as well as Supplementary Tables S1 and S2 for full LC-MS/MS data analysis of all protein hits). In whole plasma, KEGG pathways enriched in deimination and found to be common between shams and pre-motor PD models whole plasma were “complement and coagulation cascades” and “oestrogen signalling pathway” (Figure 6A,B). KEGG pathways identified to be enriched in deiminated proteins in the pre-motor PD models compared to plasma from sham-treated rats were KEGG pathways for “Parkinson’s disease”, “Alzheimer’s disease”, “Huntington’s disease” and “prion disease”, “retrograde endocannabinoid signalling”, “systemic lupus erythematosus (SLE)”, and “non-alcoholic fatty liver disease (NAFLD)” (Figure 6B). Pathways identified as enriched in deiminated proteins in sham plasma only belonged to “cholesterol metabolism”, “fat digestion and absorption”, “vitamin digestion and absorption”, “platelet activation”, “PPAR signalling pathway”, “African trypanosomiasis”, “*S. aureus* infection”, and “malaria” (Figure 6A).

**Figure 7:**
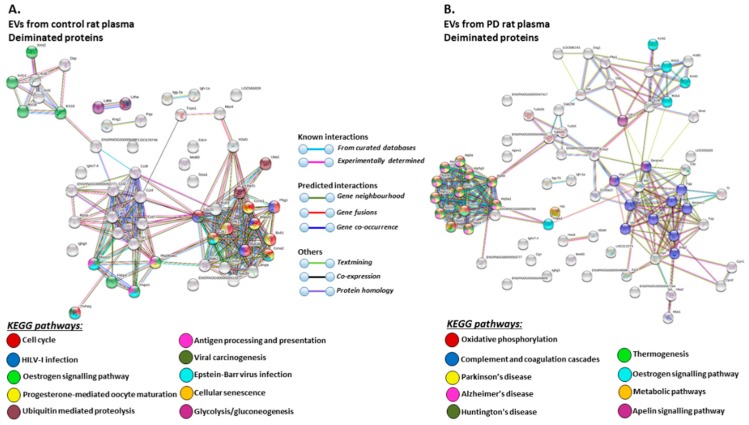
KEGG pathways for deiminated proteins in plasma-EVs of sham and pre-motor PD model rats. (**A**) KEGG pathways identified in circulating plasma-EVs of sham (control) rats are highlighted. See colour code for nodes included in the figure. (**B**) KEGG pathways identified for deiminated proteins in circulating plasma-EVs of the pre-motor PD model rats are highlighted. See colour codes for nodes and connecting lines in the figure.

The KEGG pathways identified for deiminated protein hits in plasma-EVs of the pre-motor PD models and sham-treated rats are shown in Figure 7 (Figure 7A,B; see also Table 3 and Table 4 as well as Supplementary Tables S3 and S4 for full LC-MS/MS data analysis of all protein hits). Deiminated proteins in plasma-EVs of the pre-motor PD models were enriched in KEGG pathways for “Parkinson’s disease”, “Alzheimer’s disease”, and “Huntington’s disease”, while none of these KEGG pathways were identified for deiminated protein hits in sham-treated rat plasma-EVs (Figure 7A,B). Furthermore, pathways for “oxidative phosphorylation”, “complement coagulation cascades”, “thermogenesis”, “metabolic pathways”, “*S. aureus* infection”, “gap junction”, “platelet activation”, and “apelin signalling” were only identified in plasma-EVs from the pre-motor PD models (Figure 7B). “Oestrogen signalling pathway” was common to EVs of both PD and sham-treated rat plasma. In addition, pathways enriched in deiminated proteins in sham-treated plasma-EVs and not identified in the PD models belonged to “cell cycle”, “progesterone mediated oocyte maturation”, “Epstein Barr virus infection”, “antigen processing and presentation”, “viral carcinogenesis”, “HTLV-1 infection”, “ubiquitin mediated proteolysis”, “protein processing in ER”, “cellular senescence”, “longevity regulating pathway”, “glycolysis”, and “p53 signalling pathway” (Figure 7A).

### 2.4. Deiminated Proteins are Increased in Brain Tissue of Pre-Motor PD Rats

**Figure 8:**
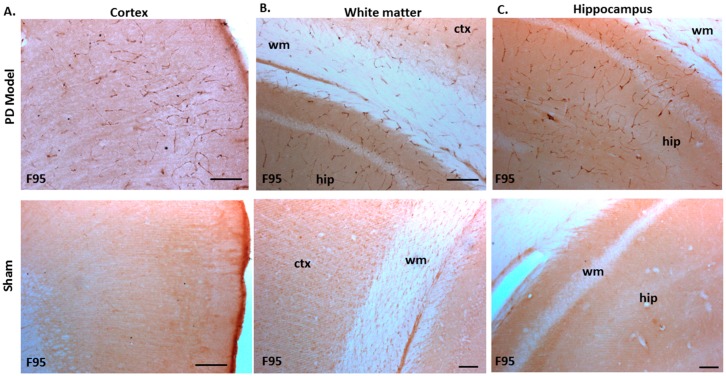

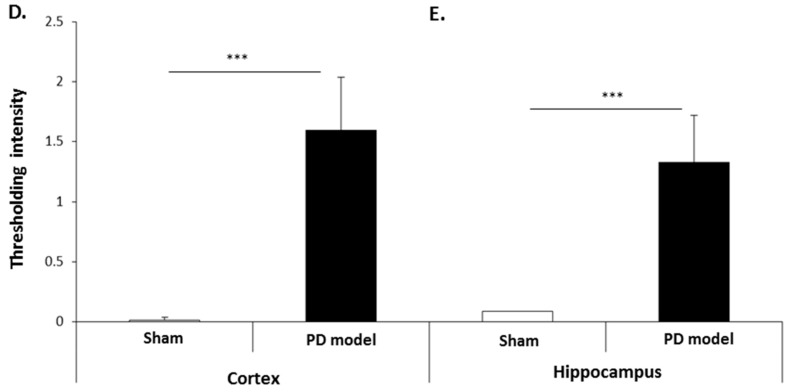
Total deiminated proteins are increased in brain tissue of pre-motor PD rats. Total deiminated protein expression was assessed by immunohistochemistry using the pan-deimination F95 antibody. (**A**) Increased protein deimination is observed in the brain vasculature in the cortex (ctx) of the pre-motor PD rat brains compared with that in the sham control brains. (**B**) Increased protein deimination is observed in white matter (wm) of the pre-motor PD rat brains compared with sham brains. (**C**) Increased protein deimination (F95 positive) is observed in the hippocampus (hip) of the pre-motor PD rat brains, compared with sham brains; the scale bars indicate 100 µm in all figures (ctx = cortex, hip = hippocampus, wm = white matter). (**D**,**E**) Thresholding intensity analysis of F95 positive staining showed a significant increase in the cortex (**D**), and the CA1 region of the hippocampus (**E**) of pre-motor PD models, compared with that in control sham brains (unpaired t-test, *** *p* < 0.001).

The pre-motor PD model brains presented an increased level of F95 immuno-positive staining compared with that of sham-treated brains. A strong positive staining for pan-deiminated proteins, by F95 detection, was observed in the cortex and hippocampus, as well as in white matter, with a highly notable positive staining in the brain vasculature (Figure 8A–C). F95 immuno-positive protein detection, assessed by thresholding intensity, showed an increase by 1.7-fold (*p* ***; *p* < 0.001) in the cortex and by 1.4-fold (*p* ***; *p* < 0.001) in the hippocampal CA1 region (Figure 8D,E), compared with shams.

**Figure 9:**
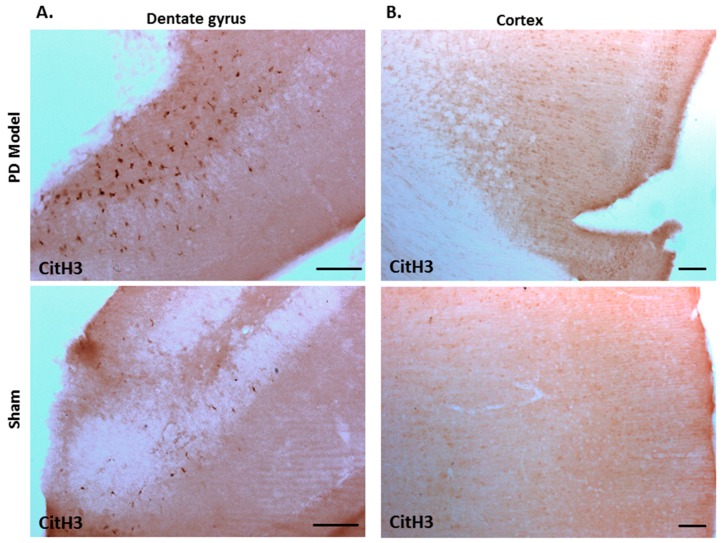
Deiminated histone H3 staining is increased in the cortex and dentate gyrus of pre-motor PD models. Immunohistochemical staining showed increased protein detection of deiminated histone H3 (citH3) in: (**A**)) the dentate gyrus; (**B**) the cortex of the pre-motor PD models, compared with those in the control sham animals. The scale bars indicate 100 µm in all figures.

Immunohistochemical staining for deiminated histone H3 (citH3) also revealed increased detection in brains of pre-motor PD models, compared with shams. CitH3 positive staining was increased in the dentate gyrus (Figure 9A), and cortex (Figure 9B) of the pre-motor PD brains, compared with shams.

**Figure 10:**
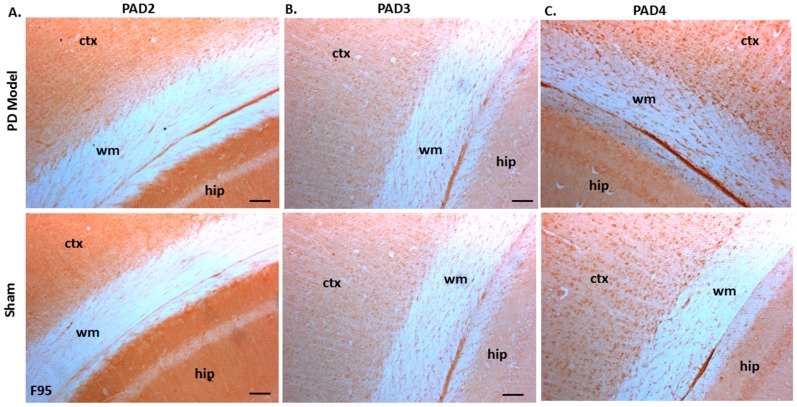
PAD2, PAD3, and PAD4 protein detection in pre-motor PD model and sham brain tissue. (**A**) PAD2 protein detection in cortex (ctx), white matter (wm) and hippocampus (hip) of sham and pre-motor PD models. (**B**) PAD3 protein detection in cortex, white matter, and hippocampus of shams and pre-motor PD rats. (**C**) PAD4 protein detection in cortex, white matter and hippocampus of shams and pre-motor PD rats. The scale bars indicate 100 µm in all figures (ctx = cortex, hip = hippocampus, wm = white matter).

Immunoreactivity for PAD2, PAD3, and PAD4 was also confirmed in both sham-treated and pre-motor PD model brains, with some elevated protein levels for PAD4 observed in the pre-motor PD model, while PAD3 showed a slight elevation and PAD2 levels were not markedly changed in the pre-motor PD models (Figure 10A–C).

## 3. Discussion

The regulation of EV release is critical for cellular communication, and particularly for the modulation of the cellular microenvironment in a range of pathologies. Mounting evidence has linked EVs to neurodegenerative diseases, indicating critical roles for EV-mediated transport of pathogenic protein and genetic cargo, including in PD [21,22]. However, roles for such EV-mediated communication in early stages of PD have received limited attention. To this end, a rat pre-motor PD model was used in this study. While this model may not display all the signs observed in PD patients, it has been shown to display some non-motor symptoms (hyposmia and cognitive impairment) in the absence of motor dysfunction. This model allows therefore a valuable insight into the time window prior to the appearance of typical PD motor symptoms and highlight potential markers of early stages of the disease. The small sample sizes in each experimental group are justified by the clear statistical differences observed between the sham animals and pre-motor models indicating good compliance of this study to the 3Rs principles.

Roles for peptidylarginine deiminases (PADs) and post-translational deimination in various neurodegenerative diseases are an area of increasing interest. Data from a human RNA-Seq transcriptome and splicing database of glia, neurones, and vascular cells of the cerebral cortex have shown that levels of PAD2 are highest in mature astrocytes, oligodendrocytes, and microglia [28]. In the majority of studies of protein deimination in neurodegenerative diseases, a focus has so far been on histological analysis of postmortem human brain samples with increased pan-protein deimination detected in both PD and Alzheimer’s disease (AD) [10,11,29,30,31]. Deimination has hitherto not been assessed in pre-motor PD and was therefore the subject of our current study as such post-translationally mediated changes may result in early onset pro-inflammatory and neurodegenerative processes and therefore offer an opportunity for early intervention. Interestingly, in the current study using a rat model of pre-motor PD, that has been shown to display non motor symptoms in the absence of motor dysfunction and neuroinflammation [32], an increase in deiminated proteins was observed in the brain vasculature, particularly in the cortex and hippocampus. Such elevated F95 positive detection observed here correlates somewhat with a previous study on human PD post-mortem brain tissue, although that study reported elevation of deiminated proteins in the substantia nigra [10]. In the current rat pre-motor PD model, substantia nigra did neither show significant changes in F95 staining (data not shown) nor in citH3 staining (Supplementary Figure S1). The increase in F95 positivity detected in other brain areas in the current study does correlate with a previous study using cellular iPSC neuronal models derived from fibroblasts from patients carrying α-synuclein triplication [15], in which an increase in total deiminated proteins was observed in vitro [9].

When assessing deiminated proteins in total plasma and plasma-EVs, KEGG pathways for AD, PD, Huntington’s disease (HD), and prion disease were found to be enriched for deiminated proteins in the pre-motor PD model, compared with shams. Protein deimination has previously been linked to AD [11,29,30,31], PD [10,12], and prion disease, including Creutzfeldt–Jakob disease (CJD) [33,34,35]. However, via F95-enrichment and protein–network analysis, the current study reveals for the first time a link between protein deimination and HD, indicative of some common pathways with other dementias, including PD, via protein deimination. Enrichment for deiminated proteins in KEGG pathways for all these neurodegenerative diseases identified in the pre-motor PD model in this current study therefore indicates putative common pathways for these neurodegenerative disorders, regulated by deimination, including some contribution of deiminated protein cargo via circulating plasma-EVs. This is in agreement with previous studies assessing KEGG pathways in AD, PD, and HD, with the aim to identify shared pathways for distinct but related diseases that may share common underlying pathogenic mechanisms [36]. Such identification may be of importance to further current understanding of these neurodegenerative disorders and for the design of new treatment strategies, especially in the early stage of the disease.

Furthermore, in the current study, KEGG pathways for “oxidative phosphorylation”, “complement coagulation cascades”, “thermogenesis”, “metabolic pathways”, “*S. aureus* infection”, “gap junction”, “platelet activation”, and “apelin signalling pathway” were only identified for F95-enriched proteins in plasma-EVs of the pre-motor PD model, and were therefore specific to the circulating plasma-EVs in the pre-motor PD model. The relevance of these pathways in neurodegeneration, and specifically in relation to what is reported in the literature for PD, is further discussed below:

Oxidative phosphorylation is a vital part of metabolism, takes place in the mitochondria, and contributes to the major source of ATP. This process also leads to the generation of reactive oxygen species (ROS) and resulting oxidative stress and bioenergetics defects, which are linked to a range of neurodegenerative disorders, including PD [37,38,39]. While oxidative stress has been shown to play significant roles both in the onset and progression of PD [40], it still remains open to debate whether the mitochondrial respiratory deficiencies observed in a range of neurodegenerative disorders are initiators or consequences of prior insult [41]. Mitochondrial dysfunction has been found to be severe in cortex and in advanced stages of PD patients, based on algorithm analysis [42]. Identification of post-translational deimination in KEGG pathways of oxidative phosphorylation that was here identified for the first time in the pre-motor PD rat model, may provide novel insights into EV-mediated transport of deiminated proteins and protein deimination mediated effects on mitochondrial dysfunction via oxidative phosphorylation pathways in the pathogenesis of neurodegenerative diseases.

The complement system is an increasingly recognised factor in neurodegenerative diseases, also with links to PD [43,44,45]. Neuroinflammation can also result in the disruption of the blood–brain barrier and therefore lead to the direct participation of the adaptive immune system [46]. While complement mediated responses are involved in chronic inflammation, the complement system forms also part of the innate and humoral responses and has been recently linked to non-motor PD [47]. Interestingly, levels of the key complement components of the alternative and classical pathways, C3 and C4, were previously reported to be lower in non-motor PD compared with healthy controls [47], while severity of disease has been related to C3 and factor H levels in PD and AD patients [48]. However, no studies have discussed putative effects of post-translational modifications of these complement components in neurodegeneration, which may be a contributing factor to altered immune and inflammatory responses in early disease stages. The post-translational deimination of several complement components, including C3, C4, and factor H, alongside a number of other complement components, has been recently identified in a range of taxa [49,50,51,52,53,54]. Indeed, in the current study, C2, C3, as well as factor H and C-type lectin were found to be deimination candidates in rat plasma, both in shams and the pre-motor PD model, while C1q, factor B, C4, C8, C9, C4-binding protein, properdin, and collectin were only identified as deiminated in the pre-motor PD model. This indicates differences in complement pathway regulation via post-translational deimination in early stages of PD. As a number of arginines may undergo post-translational deimination in all these proteins, and therefore contribute to their structural and consequently downstream functional changes, the role for deimination of complement components in early onset neurodegeneration will need to be further evaluated. Furthermore, the involvement of inflammatory factors and modified inflammatory responses and their involvement with the brain–gut axis of PD also needs consideration [55]; particularly if such factors can be identified at early stages of disease.

Thermogenesis was identified as a deimination enriched KEGG pathway. Loss of body weight and fat mass is one of the non-motor symptoms of Parkinson’s disease and has, amongst others, been related to brown fat tissue meditated thermogenesis [56]. Thermogenesis is related to mitochondrial function [57,58] and also linked to synaptic transmission and neurodegeneration [59]. Alteration in mitochondrial complexes, restricting energy output, are related to sepsis as well as PD [57] and implicated in other neurodegenerative disease such as AD, as well as being associated with diabetes [60]. Interestingly, thermogenesis is also linked to CNS activity in hibernating animals [61,62]. Deimination in thermogenesis pathways has not been identified before and provides a novel insight into putative regulation via such post-translational modifications.

Metabolic KEGG pathways were identified in this study as deiminated in plasma-EVs of pre-motor PD rats. Mitochondria are the key regulators of cellular metabolism and their dysfunction is a hallmark of PD pathogenesis [63]. Furthermore, PD has been linked to a range of inherited metabolic disorders [64]. The metabolic landscape of neurodegenerative diseases is therefore receiving increased attention [65] and alterations in metabolic pathways in PD, including in early pre-motor stages in PD, have been discussed [66]. KEGG pathways for metabolic pathways have been linked to PD development based on microarray analysis of blood samples from PD patients and matched controls [67]. The identification here of enrichment of deiminated proteins in KEGG metabolic pathways in EVs provides a novel angle of post-translational regulation of such processes.

*S. aureus* infection KEGG pathway was identified to be enriched in deiminated proteins in plasma-EVs of the pre-motor PD rats. *S. aureus* has been found to be significantly increased in the conjunctival flora in PD patients [68]. Furthermore, α-synuclein upregulation and over-expression has been correlated with elevated innate immune responses and been verified to display antimicrobial peptide-like properties and antibacterial activity against *S. aureus*, alongside other bacteria and fungi [69]. Interestingly, phagocytic activity and bactericidal activity of neutrophils have been shown to be affected in early AD [70]. However, the precise involvement of protein deimination in bacterial infection pathways and in the regulation of immune responses in PD remains to be investigated. Deimination has previously been associated with bacterial immune evasion [71], as well as with bacterial membrane vesicle release and antibiotic resistance, highlighting roles for PAD-mediated mechanisms in host–pathogen interactions [6]. This may be of some interest as the brain–gut axis relationship between the gut microflora and PD has been a topic of investigation due to gastrointestinal symptoms being one of the earliest features of PD [72]. Roles for the gut microbiota in neuroinflammation has also been hypothesised and assessed for other neurodegenerative diseases, including AD and amyotrophic lateral sclerosis (ALS) [73]. Deimination of KEGG bacterial infection pathways, or in dysbacteriosis, has not been revealed in PD or other neurodegenerative diseases in previous studies, while KEGG pathways for bacterial infection were recently identified to be enriched in deiminated proteins in glioblastoma [7].

Gap junction and platelet activation were identified in this study as KEGG pathways enriched in deiminated proteins in pre-motor PD plasma EVs. Reduced platelet activation has been described in PD [74] and furthermore a recent study has identified gap junctions and platelet activation as KEGG pathways in early PD [26]. Gap junctions are formed by pannexins and connexins and allow for exchange of ions, second messengers, and small metabolites between adjacent cells [75]. Gap junctions have critical roles in homeostasis and roles in neurodegenerative disease, including PD, have been suggested [76,77,78]. Deimination in these pathways has not been reported before and may provide novel insights into such post-translationally mediated regulation in disease and disease progression.

Apelin signalling KEGG pathway was identified to be enriched in deiminated proteins in plasma-EVs of pre-motor PD rats only. Apelin signalling is involved in a broad range of physiological functions and furthermore associated with pathologies that result from decreased vascularisation—for example ischaemia, or neovascularisations events such as tumours and retinopathes [79]. Apelin is a neuropeptide with regulatory roles of many cellular functions and involvement in a range of physiological processes including metabolic, cardiovascular function, and regulation of body fluid homeostasis [80,81]. Apelin expression has been described in endothelial cells and the CNS [82] and has also been found to be involved in neuronal dysfunction related to inflammation during ageing [83]. Modulation of apelin signalling has been discussed in a range of pathologies [84] and has recently also been associated with neurodegenerative diseases [85], including AD [86,87,88,89] and PD [90,91,92]. However, specific roles in PD remain to be further investigated. Apelin signalling has been linked to autophagy in PD, both in cellular in vitro and in vivo mouse models [90,92]. PD mouse models have shown roles for apelin in MPTP-induced damage affecting the substantia nigra, behavioral dysfunction and dopaminergic neurodegeneration [90]. Roles for apelin signalling in endoplasmic reticulum (ER) stress have also been identified [91] and neuroprotective roles of apelin signalling pathways have recently been identified in PD mouse models [93]. The current study is the first to identify apelin signalling pathways to be associated with protein deimination, and such post-translational regulation of apelin via deimination, as identified here in pre-motor PD model plasma-EVs, may play important roles in the multifaceted roles of apelin signalling.

In whole plasma, KEGG pathways identified to be enriched in deiminated proteins in the pre-motor PD model compared with plasma from sham animals were KEGG pathways for PD, AD, HD, and prion disease, retrograde endocannabinoid signalling, SLE, and NAFLD. These are further discussed below:

Retrograde endocannabinoid signalling KEGG pathways were identified in this study to be enriched in deiminated proteins in the pre-motor PD plasma only. The retrograde endocannabinoid signalling system is a lipid-based neuromodulatory system with important roles in the CNS including in physiological and neurodegenerative processes [94,95]. It has been associated to pathogenesis of PD as well as AD and ischemia [95,96,97] and suggested as a therapeutic target for treatment of a range of neurodegenerative disorders including AD, PD, HD, multiple sclerosis (MS), and ALS [98,99,100]. Putative roles for its regulation via post-translational deimination have not been previously identified and therefore bring a novel angle of modulation of endocannabinoid signalling pathways in neurodegeneration, including in early disease stages.

Systemic lupus erythematosus (SLE) KEGG pathway was identified to be enriched in deiminated proteins in plasma of the pre-motor PD rat model. SLE is a multisystemic autoimmune disease, with association to cerebrovascular diseases [101,102,103] and also with PD [104]. Furthermore, Parkinsonian syndrome has been implicated in complicating SLE [105,106]. A population-based study assessing association of PD with SLE found an inverse association between the two, with SLE patients displaying a decreased risk of subsequent PD, although this study did not include early stages of PD [107]. The deimination of SLE KEGG pathway in pre-motor PD model may provide novel insights into the previously observed interplay between SLE and PD.

NAFLD KEGG pathways were enriched in deiminated proteins in pre-motor PD model plasma-EVs. Interestingly, a link via mitochondrial dysfunction has been made between metabolic syndrome, diabetes, obesity, and non-alcoholic fatty liver disease in the progression of AD, PD, and other neurodegenerative diseases [108]. Furthermore, lipid metabolism (in particular non-alcoholic fatty liver disease pathway) and mitochondrial dysregulation have been identified as molecular pathways and putative biomarkers linked to PD [109]. Post-translational deimination is revealed for the first time for NAFLD associated KEGG pathways in this study and may be of relevance for the interplay of neurodegeneration and such comorbidities.

Recent work has assessed circulating amino acid signatures in PD patient sera, where free citrulline was found to be lower in PD than controls [110]. Furthermore, assessment of molecular signatures in circulating small EVs have been identified in relationship to mitochondria and systemic inflammation, including CD9, NDUFS3, C-reactive protein (CRP), fibroblast growth factor 21, interleukin 9, macrophage inflammatory protein 1β, and tumour necrosis factor alpha [22]. Such changes have not been assessed in pre-motor PD animal models, and interestingly in this current study, CRP is one of the deimination candidates identified in both sham and pre-motor PD model plasma. Whether the deimination levels of CRP differ between sham and pre-motor PD plasma needs to be further investigated; indeed differences in CRP deimination and between CRP forms have been recently reported in teleost fish immunity [111] but have yet to be assessed in relation to human pathologies.

Overall, deimination signatures in EVs have yet to be assessed in relation to PD or other neurodegenerative diseases. Other proteomic approaches, including Raman profiling of circulating EVs, have been used to elucidate putative links to PD progression and treatment [21]. Furthermore, proteomic analysis of serum EVs in PD patients have revealed the expression levels of seven proteins, including pigmented epithelium-derived factor, afamin, apolipoprotein D and J, are significantly increased in PD patients. Moreover, expression levels of complement C1q and protein immunoglobulins, have been reported to be decreased in PD patients [112]. Some of these proteins including C1q, immunoglobulins, and apolipoprotein A, B, and E (but not D and J) were identified to be deiminated in the pre-motor PD model, bringing a novel aspect of post-translationally mediated regulation and modification of protein function, possibly contributing to disease mechanisms. A recent study using a cellular model of PD, treated with MPP+, a well-established parkinsonian toxicant, reported that microglial EVs, when not stimulated by aggregated α-synuclein, appeared to be protective, possibly involving mitochondrial dynamics and mitochondrial fission [113]. Furthermore, deimination of Nicotinamide- N-methyltransferase (NNMT), which is implicated in several chronic diseases as well as PD, has recently been identified [114]. These previous findings, as well as our present data, further support roles for EVs and circulating deiminated proteins, including in plasma-EVs, in the involvement of PD, including in early pre-motor stages, as identified in the current study.

In the present study, the brain tissue of pre-motor PD rats showed significant increase in total deiminated proteins, as assessed by the pan-deimination F95 antibody and for deiminated histone H3. While PAD2, PAD3, and PAD4 protein expression was confirmed in the rat brains, including in the cortex, hippocampus, and white matter, there was no significant difference in PAD isozyme protein levels, although some elevated levels were noted for PAD4 and to a lesser level for PAD3 protein in the pre-motor PD brains. Their deiminated protein products showed though a significant and marked increase in the pre-motor PD brains, particularly in the cortex, white matter, and hippocampus, while deiminated histone H3 was increased in the dentate gyrus as well as in the cortex. As protein deimination is a result of PAD activation, increased levels of PAD proteins per se would not necessarily be expected at this stage, while an increased production of deiminated protein products would be the result of PAD activation, leading to increased production of deiminated protein products. As dysregulation of calcium homeostasis is a known contributor to a number of neurodegenerative diseases, including in PD [115,116], and PADs are calcium activated enzymes, an increase in protein deimination would be one of such downstream factors. In previous studies on post-mortem PD brain samples, increased levels of total protein deimination and deimination-positive extracellular plaques have indeed been reported [10]. Furthermore, the presence of mutated misfolded α-synuclein protein has also been related to increased protein deimination [12]. Supporting those post-mortem studies, in vitro studies using iPSC derived neuronal models carrying α-synuclein triplication, revealed increased protein deimination levels, although specific target proteins of deimination were not further assessed [9].

A hitherto unrecognised contribution of deiminated proteins to the inflammatory responses, which previously have been shown in the pre-motor PD rat model [32], has to also be considered via the release of deiminated neuronal proteins from necrotic neurons, as well as via the circulation of deiminated proteins in the cerebrospinal fluid, which can contribute to progressive pathology due to generation of autoantibodies [117]. Furthermore, the significant increase observed in F95-positive brain vasculature of the pre-motor PD model, in particular in the cortex and hippocampus, and to some extent in the white matter, may also affect both local and systemic EV release and contribute to the spread and progression of pathology. Deiminated proteins also expose neo-epitopes which, in addition to leakage of deiminated proteins from dying cells, can further contribute to neuro-inflammatory responses. This also includes histone deimination which, in addition to gene regulatory effects, may cause extracellular trap formation which can contribute to local tissue damage [118]. Increased levels of deiminated histone H3 were here observed in the dentate gyrus as well as in cortex of the pre-motor PD brains. Such increase in citH3 positive detection may be of considerable importance in the neuroinflammatory environment; indeed, previous studies of CNS injury, including via hypoxic damage, have shown that pharmacological inhibition of PADs, and associated reduction of citH3, correlates with neuroprotective effects [119,120,121].

Recent work has emphasised the association of selected microRNAs to PD [27,122,123] and roles for microRNAs in neuroinflammation have received increased attention [25,124]. As neuroinflammation is thought to be a facilitator of PD pathogenesis, and was observed in the pre-motor PD model used in this study [32,125], we assessed changes in relative expression levels of three inflammatory and hypoxia associated microRNAs: miR21, miR155, and miR210. These microRNAs have previously been related to neurodegenerative diseases, including PD, although none of these microRNA (miRs) have been assessed, or associated with, pre-motor PD before the current study. All the microRNAs were here found to be significantly increased in circulating plasma-EVs of the pre-motor PD model. Known roles for these microRNAs in relation to neurodegeneration, including PD, are discussed below:

miR21 is a key regulator of inflammation [126], also associated with oxidative stress [127] and involved in neuroinflammatory regulation [128,129,130]. miR21 has been found to be upregulated in multiple sclerosis (MS), although specific functions have not been identified [131]. In PD, miR21 has been associated to autophagy [132]. Moreover, miR21 has been identified as a biomarker in plasma/serum for AD, prion disease, as well as traumatic brain injury [24,124,133,134], where it has also been found to be increased in EVs [135]. The current study is the first one to identify elevated miR21 as a marker of pre-motor PD.

miR155 has previously been identified as a contributor to the induction of neuroinflammation [25,136] and to increase blood–brain barrier (BBB) permeability [137]. In PD, miR155 has been linked to microglial inflammatory response induced by α-synuclein [23]. It has also been associated with various other neuroinflammatory disorders including MS, where it has been identified as a biomarker [138]. mir155 is also found to be elevated in AD and to contribute to neuroninflammation in this disease [139] and identified as a biomarker [124,133]. In PD, miR155 has been implicated in mitochondrial regulation in dopaminergic cell death in later stage [140], however this study presents a link between miR155 and pre-motor PD for the first time.

miR210 is a hypoxia-related microRNA, also linked to inflammation, and is induced under hypoxic conditions. It plays key roles in mitochondrial metabolism, apoptosis, cell proliferation, and the DNA damage response [141,142,143,144]. miR210 has been associated with mitochondrial dysfunction and oxidative stress in relation to neurodegenerative diseases [145,146]. It has previously been linked to PD in cell culture models, assessing environmental neurotoxicant mangane, in which miR210 was identified in EVs [147]. The interplay between oxidative stress and microRNAs has been shown in a number of neurodegenerative diseases including AD, PD, HD, and ALS [148]. Oxidative stress has indeed been suggested to play key roles in PD, although some parameters from clinical studies have been inconsistent [149]. Therefore, the identification in the current study of miR210 being significantly elevated in the pre-motor PD model indicates a hitherto unrecognized contribution by EV-mediated transport of this microRNA in early stages of PD.

Modifications of EV-mediated export of misfolded proteins, DNA, RNA, miRNAs, enzymes, and other EV cargo may be of considerable importance already in early stages of PD, and in neurodegenerative disease progression, and contribute to pathology. The transport of EV transcytosis across the BBB and associated release of EV cargo from brain endothelial cells into the systemic circulation has for example been suggested [150]. This correlates with strongly F95-positive vasculature observed in the pre-motor PD brains in the current study, which could also contribute to increased EV release from these sites. Therefore, the identification of early changes, such as EV numbers released and associated changes in specific EV microRNA and deiminated protein cargo, as identified here in circulating EVs in the rat pre-motor PD model, may offer novel markers indicative of early stages of pre-motor PD and be developed into useable screening tools.

## 4. Materials and Methods

### 4.1. Induction of the Rat Model of Pre-Motor PD

All procedures were approved by the Bloomsbury ethical committee and the Home Office and followed the British Home Office regulations with regard to the Animal Scientific Procedures Act 1986 (PPL PP3144142). Male Sprague-Dawley rats (200–250 g—Charles River Laboratories, U.K.) were kept under constant conditions of humidity (40–60%), temperature (18–22 °C), and a 12 h light–dark cycle. The induction of the pre-motor model was carried out as previously described [32]. In brief, intraperitoneal administration of either N-(2-chloroethyl)-N-ethyl-2-bromobenzylamine (DSP-4, Sigma-Aldrich; a noradrenergic neurotoxin- pre-motor model) at a dose of 25 mg/kg, or sterile saline (sham animals) was performed 4 days prior to dopaminergic neurotoxin insult with 6-hydroxydopamine (6-OHDA). Bilateral striatal injections of either 6-OHDA (Sigma-Aldrich—dissolved in saline solution containing 0.9% ascorbic acid- pre-motor model) or saline containing 0.9% ascorbic acid for sham animals were then performed using the following coordinates from the atlas of Paxinos and Watson (1982), from Bregma: AP +1.0 mm, ML +3.0 mm, DV −6.5 mm. Animals were anaesthetised using isoflurane (5% v/v in O2 for induction and 2% v/v in O2 for maintenance) delivered through a fitted nose mask and rats were secured to a stereotaxic frame using blunt ear bars (David Kopf Instruments, Bethesda, MD, USA). Each animal received 15 μg of 6-OHDA per striatum (or vehicle) at a flow rate of 1 μL/min-1. Daily monitoring of the animals was performed following the surgical procedures. Rats were divided into two experimental groups: sham-treated animals (*n* = 3) and toxin-treated model (pre-motor model—*n* = 3). This pre-motor PD model was shown to present non-motor symptoms in the absence of motor dysfunction in a previous study [32]. The pre-motor PD models did not present any motor dysfunction prior to culling.

### 4.2. Immunohistochemistry

All histological procedures have been previously described [32,151]. Sham animals and pre-motor PD models were anaesthetised by inhalation of isoflurane and intraperitoneal injection of Euthatal (Merial, Harlow, UK) (60 mg/kg) and perfused transcardiacally with ice-cold oxygenated artificial cerebrospinal fluid (ACSF) containing in mM: 124 NaCl, 25.5 NaHCO_3_, 3.3 KCl, 1.2 KH_2_PO_4_, 1 MgSO_4_, 2.5 CaCl_2_, 15 mM D-Glucose equilibrated with 95% O_2_/5% CO_2_. Brains were removed and fixed overnight (4% paraformaldehyde, 0.2% saturated picric acid solution, 0.025% glutaraldehyde solution in 0.1 M Phosphate buffer). Fifty micrometers coronal sections containing cortex and hippocampus were cut with a vibratome (Agar Scientific Ltd, Stansted, UK). One in eight sections were collected and 4–5 slices per animal were used per staining. Sections were incubated first in 1% H_2_O_2_ for 30 min and then in 1% sodium borohydride (NaBH_4_) for 30 min to decrease background staining and then blocked in 10% normal goat serum (NGS) for another 30 min to block non-specific antibody binding. Sections were incubated overnight at 4 °C in the respective primary antibodies diluted in Phosphate Buffer Saline (PBS) containing 0.5% Triton X-100 (Sigma-Aldrich, Haverhill, UK): Mouse anti-peptidyl-citrulline F95 (for total deiminated proteins, MABN328, Merck, diluted 1:200), rabbit anti-Histone H3 (citrulline R2 + R8 + R17, ab5103, Abcam, diluted 1:200), rabbit anti-PAD2 (ab50257, Abcam; diluted 1:200), rabbit anti-PAD3 (ab50246; diluted 1:200) and rabbit anti-PAD4 (ab50332; diluted 1:200). Biotinylated labelled secondary anti-mouse IgM (Vector Laboratories—diluted 1:100) or anti-rabbit IgG (Diluted 1:500—Vector laboratories) were applied for 6 h. Sections were then washed and incubated in ABC (Vector Laboratories) overnight, washed, and then incubated in 3, 3′ diaminobenzidine (DAB- Sigma Aldrich) for 20 min. H_2_O_2_ was added to the DAB solution to allow the reaction until the filled cells were sufficiently labelled. Sections from the two groups were processed together and the DAB reaction was stopped at the same time to allow comparison between the shams and pre-motor models. Sections were placed onto Superfrost slides, dehydrated, cleared with Histoclear, and mounted using DPX (Sigma Aldrich). Levels of F95 immunohistochemical staining were measured by quantitative thresholding image analysis as previously described (Rahim et al., 2012; Sancandi et al., 2018). Images of both cortex and hippocampal CA1 in each section were captured using a DMR microscope and Leica Application Suite V4 (Leica Microsystems, Wetzlar, Germany) at 5× magnification with constant light intensity, microscope calibration, and video camera settings. Images were analysed using Image-Pro Premier (Media Cybernetics, Cambridge, UK) by measuring immunoreactivity using a constant threshold that was applied to all images for each respective antigen. Data are presented as the mean percentage area of immunoreactivity ± SEM.

### 4.3. EV isolation and Quantification by Nanoparticle Tracking Analysis

Plasma was isolated from blood collected from the heart of the anaesthetized rats via cardiac puncture prior to perfusion (described above). Two aliquots of 1.5 mL of blood were collected from each animal and plasma was isolated by centrifugation at 2000 g for 10 min, immediately aliquoted and frozen at −80 °C. EV isolation was carried out according to previously established protocols [5,7,54] as well as the recommendations of the International Society of Extracellular Vesicle Research (ISEV) [152]. Plasma aliquots were thawed before EV isolation and differential centrifugation was carried out by adding 100 µL plasma to 400 µL Dulbecco’s PBS (DPBS) and centrifuged at 4000 g for 30 min at 4 °C for removal of aggregates and apoptotic bodies. Next, centrifugation of the collected supernatant was carried out for 1 h at 4 °C at 100,000 g. The supernatant was then discarded and the isolated EV pellets were resuspended in ice-cold DPBS and centrifuged again at 100,000 g for 1 h at 4 °C. Thereafter, the final EV enriched pellet was resuspended in 100 μL sterile EV-free PBS. Nanoparticle tracking analysis (NTA) was carried out to quantify the EVs, using the NS300 Nanosight (Malvern Panalytical Ltd, Malvern, UK), equipped with a sCMOS camera and a 405 nm diode laser. EV samples were diluted 1:100 in sterile-filtered EV-free DPBS before application to the Nanosight. Particle numbers in the field of view were maintained within the rage of 40–60. The camera settings were set according to the manufacturer’s instructions (Malvern) at level 10 for recording of five 60 s videos per sample. For post-analysis of the recorded videos, the detection threshold was set at 5 and the obtained replicate histograms were averaged for each sample.

### 4.4. Western Blotting Analysis for EV Characterisation

For western blotting analysis, the plasma-EVs were re-constituted in 2 × Laemmli sample buffer containing 5% β-mercaptoethanol (BioRad, Kidlington, UK) and heated for 5 min at 100 °C. The proteins were separated on 4–20% Mini-Protean TGX protein gels (BioRad) by sodium dodecyl sulfate polyacrylamide gel electrophoresis (SDS-PAGE), followed by semi-dry western blotting. Ponceau S staining (Sigma, U.K.) was used to assess even transfer to nitrocellulose membranes (0.45 μm, BioRad), which were thereafter blocked for 1 h at room temperature (RT) in 5% bovine serum albumin (BSA) (Sigma) in Tris buffered saline (TBS) containing 0.1% Tween20 (TBS-T). Primary antibody incubation was carried out overnight at 4 °C using antibodies for two EV-specific markers: CD63 (ab68418; Abcam, Cambridge, UK; diluted 1:1000 in TBS-T) and Flotillin-1 (Flot-1; ab41927; Abcam; diluted 1:2000 in TBS-T). Thereafter, membranes were washed in TBS-T and incubated at RT for 1 h with HRP-conjugated anti-rabbit IgG secondary antibodies (BioRad, diluted 1:3000 in TBS-T). The membranes were washed for five times 10 min in TBS-T, followed by a final wash in TBS without Tween20. Protein bands were visualised using enhanced chemiluminescence (ECL; Amersham, U.K.) and the UVP BioDoc-ITTM System (Thermo Fischer Scientific, Hemel Hempstead, UK).

### 4.5. EV Characterisation by Transmission Electron Microscopy

TEM imaging of EVs was carried out according to previously described protocols [7,53]. The plasma-EVs were resuspended in 100 mM sodium cacodylate buffer (pH 7.4). One drop (~3–5 μL) of the EV suspension was placed onto a grid carrying a carbon support film, which previously had been glow discharged. After the suspension had partly dried, the grid was placed on to a drop of a 2.5% glutaraldehyde solution in 100 mM sodium cacodylate buffer (pH 7.4) for 1 min and thereafter washed by touching it to the surface of three drops of distilled water. Excess water was carefully removed using a filter paper. A drop of 2% aqueous Uranyl Acetate stain (Sigma-Aldrich) was next applied to the grid. Following 1 min, any excess stain was removed using a filter paper. The grid was dried at room temperature, whereafter the samples were imaged in TEM using a JEOL JEM 1400 transmission electron microscope (JEOL, Tokyo, Japan), which was operated at 80 kV at a magnification of 30,000 to 60,000. Digital images of EVs were recorded with an AMT XR60 CCD camera (Deben UK Limited, Bury Saint Edmunds, UK).

### 4.6. Analysis of microRNAs miR21, miR155, and miR210 in Plasma-EVs

For assessment of microRNA cargo in plasma-derived EVs of the pre-motor PD models and sham treated rats, EVs were isolated as described above and further processed for RNA isolation, cDNA translation, and assessment for expression of miR21, miR155, and miR210, according to previously described protocols [7]. First, RNA was extracted using Trizol (Sigma Aldrich, Gillingham, UK) and thereafter the RNA concentration and purity were measured by NanoDrop Spectrophotometer at 260 nm and 280 nm absorbance. The RNA was then reverse transcribed to cDNA, using the qScript microRNA cDNA Synthesis Kit (Quantabio, Beverly, MA, USA), following the manufacturer’s instructions. The resulting cDNA was utilised for assessment of relative expression levels of miR21, miR155, and miR210. Reference RNAs used for normalisation of miR expressions were U6 and hsa-let-7a-5p. MystiCq microRNA qPCR primers for miR21 (hsa-miR-21-5p), mir155 (hsa-miR-155-5p), and miR210 (hsa-miR-210-5p), all obtained from Sigma (U.K.), were used together with the PerfeCTa SYBR^®^ Green SuperMix (Quantabio). The sequences for U6-snRNA primers were as follows: U6 forward, 5′-GCTTCGGCAGCACATATACTAAAAT-3′ and hsa-let-7a-5p forward 5′-CCGAGCTGAGGTAGTAGGTTGTATA-3′ reverse 5′-CGCTTCACGAATTTGCGTGTCAT-3′ for both. The following thermocycling conditions were applied: Denaturation: 95 °C for 2 min; followed by 40 cycles at 95 °C for 2 s and 60 °C for 15 s; extension was carried out at 72° C for 15 s. Expression levels of miR21, miR155, and miR210 were thereafter normalized to that of U6, according to the 2ΔΔCT method [153]. Each experiment was repeated in biological and technical triplicates.

### 4.7. Assessment of KEGG Pathways for Deiminated Proteins in Plasma and Plasma-EVs

F95 enrichment of deiminated proteins was carried out according to previously described protocols [7,53,54]. Rat plasma (a pool of 3 × 30 μL, using 30 μL aliquots from 3 individual animals per experimental group) and plasma-EVs (a pool of 3 × 30 μL extracted EVs from 3 individual animals per experimental group) respectively, were added to mini-prep sepharose columns in the presence of the pan-deimination F95 antibody (MABN328, Merck, Feltham, UK; [154]) for immunoprecipitation of total deiminated proteins, using the Catch and Release^®^ v2.0 Immunoprecipitation Kit according to the manufacturer’s instructions (Merck). F95-enrichment was carried out overnight at 4 °C on a rotating platform and the F95 bound proteins were thereafter eluted using denaturing elution buffer, according to the manufacturer’s instructions (Merck). F95-enriched eluates from the plasma and plasma-EVs were thereafter analysed both by SDS-PAGE electrophoresis followed by silver staining as well as by liquid chromatography with tandem mass spectrometry (LC-MS/MS; Cambridge Proteomics, Cambridge, UK), according to previously described methods [7,53]. For LC-MS/MS, the F95-enriched eluates were first run 0.5 cm into a 12% TGX gel (BioRad) and thereafter cut out as one band each, respectively. The 1D gel bands were then transferred into a 96-well PCR plate. The gel-bands were cut into 1 mm^2^ pieces, destained, reduced using DTT, and alkylated using iodoacetamide, and thereafter digested with trypsin overnight at 37 °C. Thereafter, the supernatant was pipetted into a sample vial, following by loading onto an autosampler for automated LC-MS/MS analysis using a Dionex Ultimate 3000 RSLC nanoUPLC (Thermo Fisher Scientific Inc., Waltham, MA, USA) system in conjunction with a QExactive Orbitrap mass spectrometer (Thermo Fisher Scientific Inc., Waltham, MA, USA). Peptide separation was carried out using reverse-phase chromatography (flow rate 300 nL/min) and a Thermo Scientific reverse-phase nano Easy-spray column (Thermo Scientific PepMap C18, 100A pore size, 2 µm particle size, 75 µm i.d. ×50 cm length). The peptides were loaded onto a pre-column (Thermo Scientific PepMap 100 C18, 5 µm particle size, 100A pore size, 300 µm i.d. ×5 mm length) from the Ultimate 3000 autosampler together with 0.1% formic acid for 3 min (flow rate 10 µL/min). Thereafter, the column valve was switched for elution of peptides from the pre-column onto the analytical column. Solvent A was water containing 0.1% formic acid, while solvent B contained 20% water and 80% acetonitrile, as well as 0.1% formic acid. A linear gradient of 2–40% B was employed for 30 min. The LC eluent was sprayed into the mass spectrometer using an Easy-Spray source (Thermo Fisher Scientific Inc.). The m/z values of all eluting ions were measured in an Orbitrap mass analyzer, which was set at a resolution of 70,000, while scanning was performed between m/z 380–1500. Data dependent scans (selecting top 20) were employed for automatic isolation and generation of fragment ions by higher energy collisional dissociation (HCD, NCE:25%) using the HCD collision cell. The resulting fragment ions were measured using the Orbitrap analyser, which was set at a resolution of 17,500. Both singly charged ions as well as ions with unassigned charge states were excluded from selection for MS/MS. A dynamic exclusion window of 20 sec was also applied. The data was processed post-run using Protein Discoverer (version 2.1., Thermo Scientific). In brief, MS/MS data was converted to mgf files which were submitted Mascot (Mascot search algorithm; Matrix Science, London, UK). Search for hits was carried out against the UniProt Rattus_norvegicus_20181203 (31,558 sequences; 17,280,660 residues) database as well as common contaminant sequences database (123 sequences; 40,594 residues). Peptide and fragment mass tolerances were respectively set at 20 ppm and 0.1 Da. The threshold value for significance was set at *p* < 0.05, while the peptide cut-off score was set at 20. To identify protein–protein interaction networks for deiminated proteins identified in whole plasma and plasma-EVs, respectively, STRING analysis (Search Tool for the Retrieval of Interacting Genes/Proteins; https://string-db.org/) was used. The protein networks were built as follows: function applied in STRING was “search multiple proteins”, the species database chosen was “Rattus norvegicus”, and “basic settings and medium confidence were applied. Colour lines between the nodes indicate the following evidence-based interactions for network edges: “known interactions” (these are based on curated databases, experimentally determined), as well as “predicted interactions” (these are based on gene neighbourhood, gene fusion, gene co-occurrence, or via text mining, co-expression, or protein homology).

### 4.8. Silver Staining

Following electrophoresis under reducing conditions as described above, using SDS-PAGE 4–20% TGX gels (BioRad), the F95-enriched eluted proteins from rat plasma and plasma-EVs were stained by silver staining. Staining was carried out using the BioRad Silver Stain Plus Kit (1610449, BioRad, UK), according to the manufacturer’s instructions (BioRad).

### 4.9. Statistical Analysis

All graphs were prepared and statistical analysis was performed using GraphPad Prism version 7 (GraphPad Software, San Diego, CA, USA). The experiments were repeated in triplicates, all histograms represent mean of data, while the error bars indicate standard deviation (SD). Significant differences were considered as *p* ≤ 0.05, following Student’s t-test. The NTA curves were generated by the NanoSight 3.0 software (Malvern, UK) with the black line representing the mean of the 5 repetitive readings per sample and the red line representing standard error (+/−).

## 5. Conclusions

In summary, the findings of the current study highlight PAD-mediated communication in early pre-motor stages of PD, both with respect to cerebrovascular changes as well as post-translational changes in protein networks relating to neurodegenerative diseases in plasma and EV-cargo. Changes of EV-mediated microRNA export were also verified, with increased expression of inflammatory and hypoxia related microRNAs in circulating plasma-EVs in the pre-motor PD model. Overall, an increase in F95 positive proteins, both in brain tissue and plasma, as well as in circulating plasma-EVs observed here in the pre-motor PD model, highlights hitherto under-recognized roles for these parameters in early stages of PD. Our findings emphasise the need to further assess and refine protein deimination and EV related microRNA and deimination biomarkers for early pre-motor PD diagnosis.

## Figures and Tables

**Table 1:** Deiminated proteins in sham-treated rat plasma (*Rattus norvegicus*), as identified by F95-enrichment and liquid chromatography mass spectrometry (LC-MS/MS) analysis. Deiminated proteins were isolated from sham-treated rat plasma by immunoprecipitation using the pan-deimination F95 antibody. The resulting F95-enriched eluate was then analysed by LC-MS/MS and peak list files submitted to mascot. *Rattus norvegicus* species-specific peptide sequence hits are listed, showing number of sequences for protein hits and total score. Blue highlighted rows indicate protein hits identified in whole plasma of sham-treated rats, but not identified as deiminated in plasma of the pre-motor PD model.

Protein Name	Symbol	Sequences	Total Score (*p* < 0.05) ^ⱡ^
Serum albumin	A0A0G2JSH5_RAT	38	2280
Serum albumin	ALBU_RAT	37	2215
Alpha-1-macroglobulin	A1M_RAT	34	1890
Alpha-1-inhibitor 3	A1I3_RAT	25	1615
Complement C3	M0RBF1_RAT	30	1611
Similar to histidine-rich glycoprotein	A0A0G2K9Y5_RAT	28	1561
Murinoglobulin-1	D4A6E3_RAT	22	1330
Kininogen-1	KNG1_RAT	20	1112
Ac1873	Q7TQ70_RAT	21	1008
Serotransferrin	TRFE_RAT	18	936
Isoform LMW of Kininogen-1	KNG1_RAT	15	860
Ig gamma-2C chain C region	IGG2C_RAT	13	739
Isoform 2 of Fibrinogen beta chain	FIBB_RAT	14	545
Ig gamma-2A chain C region	IGG2A_RAT	8	520
Isoform Gamma-A of Fibrinogen gamma chain	FIBG_RAT	10	516
Carboxypeptidase N catalytic chain	CBPN_RAT	8	460
Uncharacterized protein	A0A0G2JZW1_RAT	8	446
C-reactive protein	CRP_RAT	6	432
Fibronectin	A0A096P6L8_RAT	9	404
Ig kappa chain C region, A allele	KACA_RAT	6	383
Uncharacterized protein	A0A0G2JVP4_RAT	7	341
Uncharacterized protein	A0A0G2K477_RAT	6	333
Hemoglobin subunit beta-1	HBB1_RAT	6	319
Apolipoprotein E	A0A0G2K151_RAT	7	316
Uncharacterized protein	D4ACR1_RAT	4	300
Hemoglobin subunit alpha-1/2	HBB1_RAT	6	295
Uncharacterized protein	D4ACR1_RAT	4	274
Kininogen-1	A0A0G2JVQ5_RAT	6	251
Carboxypeptidase N subunit 2	F1LQT4_RAT	6	250
Ig gamma-2B chain C region	IGG2B_RAT	5	237
Hemopexin	HEMO_RAT	6	231
Ceruloplasmin	A0A0G2K9I6_RAT	6	220
Hemoglobin subunit beta-2	HBB2_RAT	4	216
Uncharacterized protein	M0RA79_RAT	3	213
Keratin, type II cytoskeletal 5	A0A0G2K509_RAT	3	210
Alpha-1-antiproteinase	A0A0G2JY31_RAT	3	209
Uncharacterized protein	A0A0G2K290_RAT	3	203
Keratin, type II cytoskeletal 6A	K2C6A_RAT	3	198
Ab1-233	A0A0G2JXK5_RAT	4	197
Uncharacterized protein	F1LVL4_RAT	3	186
Keratin, type I cytoskeletal 14	K1C14_RAT	3	181
Uncharacterized protein	F1M3 × 3_RAT	4	177
Kallikrein B, plasma 1	Q5FVS2_RAT	3	175
Complement C2	Q6MG73_RAT	4	175
Uncharacterized protein	A0A0G2JY98_RAT	3	172
Haptoglobin	A0A0H2UHM3_RAT	3	151
Selenoprotein P	SEPP1_RAT	4	151
Apolipoprotein A-I	APOA1_RAT	2	134
Serine protease inhibitor A3K	SPA3K_RAT	4	130
Uncharacterized protein	F1LXY6_RAT	2	123
Uncharacterized protein	A0A0G2JW41_RAT	1	115
Keratin 16	Q6IFU9_RAT	3	115
Ig lambda-2 chain C region	LAC2_RAT	2	112
Uncharacterized protein	A0A0G2K4K2_RAT	3	111
Coagulation factor XII	A0A0H2UI19_RAT	3	110
Inter-alpha-trypsin inhibitor heavy chain family, member 4	D3ZFC6_RAT	2	105
Uncharacterized protein	F1LZH0_RAT	2	93
Uncharacterized protein	F1M0U4_RAT	2	93
RCG21066	D3ZJW6_RAT	2	86
Uncharacterized protein	M0RAB8_RAT	1	85
Uncharacterized protein	M0R816_RAT	1	83
C-type lectin domain family 11 member A	CLC11_RAT	2	80
Complement factor H	F1M983_RAT	2	79
Beta-globin	Q62669_RAT	2	71
Uncharacterized protein	A0A0G2JV42_RAT	2	62
Uncharacterized protein	F1LM19_RAT	1	53
Inter-alpha-trypsin inhibitor heavy chain 2	D3ZFH5_RAT	2	53
Uncharacterized protein	F1M5L5_RAT	2	52
Fetub protein	Q6IRS6_RAT	1	45
Uncharacterized protein	A0A0G2JWX0_RAT	1	45
Uncharacterized protein	A0A0G2JTG4_RAT	1	42
Ubiquitin carboxyl-terminal hydrolase isozyme L1	UCHL1_RAT	1	41
Uncharacterized protein	A0A0G2JUY3_RAT	1	41
Apolipoprotein A-IV	A0A0G2JVX7_RAT	1	39
Extracellular calcium-sensing receptor	CASR_RAT	1	37
Uncharacterized protein	F1LZ11_RAT	1	36
Uncharacterized protein	M0R9U2_RAT	1	34
Golgi apparatus protein 1	G3V8G5_RAT	1	33

^ⱡ^ Ions score is −10*Log(P), where P is the probability that the observed match is a random event. Individual ions scores > 31 indicated identity or extensive homology (*p* < 0.05). Protein scores were derived from ions scores as a non-probabilistic basis for ranking protein hits.

**Table 2:** Deiminated proteins in pre-motor PD rat plasma (*Rattus norvegicus*), as identified by F95-enrichment and LC-MS/MS analysis. Deiminated proteins from pre-motor PD rat plasma were isolated by immunoprecipitation using the pan-deimination F95 antibody. The resulting F95-enriched eluate was then analysed by LC-MS/MS and peak list files submitted to mascot. *Rattus norvegicus* species-specific peptide sequence hits are listed, showing number of sequences for protein hits and total score. Pink highlighted rows indicate protein hits identified in pre-motor PD plasma but not sham-treated rat plasma.

Protein Name	Symbol	Sequences	Total Score (*p* < 0.05) ^ⱡ^
Complement C3	M0RBF1_RAT	89	5530
Alpha-1-macroglobulin	A1M_RAT	74	4911
Alpha-1-inhibitor 3	A1I3_RAT	59	3845
Fibronectin	A0A096P6L8_RAT	64	3722
Murinoglobulin-1	MUG1_RAT	57	3618
Serum albumin	ALBU_RAT	56	3564
Serum albumin	A0A0G2JSH5_RAT	56	3550
Fibrinogen beta chain	FIBB_RAT	53	3399
Serotransferrin	TRFE_RAT	53	3384
Ac1873	Q7TQ70_RAT	56	3317
Isoform Gamma-A of Fibrinogen gamma chain	FIBG_RAT	31	2185
Similar to histidine-rich glycoprotein	A0A0G2K9Y5_RAT	38	2137
Kininogen-1	KNG1_RAT	33	1981
Complement C2	Q6MG73_RAT	30	1875
Ceruloplasmin	A0A0G2K9I6_RAT	32	1816
Complement factor H	G3V9R2_RAT	28	1570
Kallikrein B, plasma 1	Q5FVS2_RAT	27	1552
Ig gamma-2C chain C region	IGG2C_RAT	21	1447
Complement factor B	A0A0U1RRP9_RAT	24	1405
Hemopexin	HEMO_RAT	25	1388
Keratin, type II cytoskeletal 5	K2C5_RAT	24	1284
Uncharacterized protein	G3V9J1_RAT	22	1197
Inter alpha-trypsin inhibitor, heavy chain 4	Q5EBC0_RAT	22	1192
Carboxypeptidase N catalytic chain	CBPN_RAT	18	1172
Uncharacterized protein	A0A0G2JVP4_RAT	18	1133
Ig gamma-2A chain C region	IGG2A_RAT	16	1108
Complement C4	CO4_RAT	20	1070
Carboxypeptidase N subunit 2	F1LQT4_RAT	19	1042
Plasminogen	PLMN_RAT	20	1001
Apolipoprotein A-IV	APOA4_RAT	17	989
Uncharacterized protein	A0A0G2JZW1_RAT	13	980
Inter-alpha trypsin inhibitor, heavy chain 1	B2RYM3_RAT	18	960
Complement C5	A0A096P6L9_RAT	21	959
Apolipoprotein E	A0A0G2K151_RAT	13	813
Keratin, type I cytoskeletal 14	K1C14_RAT	14	779
Complement C4B (Chido blood group)	Q6MG90_RAT	13	769
Ab1-233	A0A0G2JYK0_RAT	14	760
Inter-alpha-trypsin inhibitor heavy chain 2	D3ZFH5_RAT	15	755
Uncharacterized protein	F1LTJ5_RAT	14	735
Serine protease inhibitor A3L	SPA3L_RAT	14	734
Serine protease inhibitor A3K	SPA3K_RAT	12	685
Keratin, type I cytoskeletal 42	K1C42_RAT	12	660
Uncharacterized protein	A0A0G2K477_RAT	11	656
Haptoglobin	A0A0H2UHM3_RAT	14	650
Keratin, type I cytoskeletal 17	A0A0G2K9Q9_RAT	14	634
Alpha-1-antiproteinase	A0A0G2JY31_RAT	11	613
Inter-alpha-trypsin inhibitor heavy chain H3	D3ZBS2_RAT	13	610
Selenoprotein P	SEPP1_RAT	9	590
C-type lectin domain family 11 member	CLC11_RAT	12	587
Uncharacterized protein	M0RA79_RAT	10	571
Apolipoprotein A-I	APOA1_RAT	10	558
C-reactive protein	CRP_RAT	7	554
Coagulation factor XII	A0A0H2UI19_RAT	11	550
Ig kappa chain C region, A allele	KACA_RAT	7	536
Uncharacterized protein	F1M3X3_RAT	9	536
Insulin-like growth factor binding protein, acid labile subunit, isoform CRA_b	F1LRE2_RAT	9	529
Kininogen-1	A0A0G2JVQ5_RAT	9	524
Uncharacterized protein	F1LXY6_RAT	7	507
Keratin 16	Q6IFU9_RAT	9	504
Uncharacterized protein	A0A0G2K828_RAT	7	500
Keratin, type II cytoskeletal 75	A0A0H2UHH5_RAT	9	496
Uncharacterized protein	D4ACR1_RAT	8	471
Apolipoprotein B-100	F1M6Z1_RAT	12	457
Keratin, type II cytoskeletal 6A	K2C6A_RAT	9	446
Keratin, type II cytoskeletal 8	K2C8_RAT	8	435
Apolipoprotein H	Q5I0M1_RAT	9	435
Uncharacterized protein	D3ZZ08_RAT	7	433
Ig gamma-2B chain C region	IGG2B_RAT	8	433
Carboxylesterase 1C	EST1C_RAT	8	431
Hemoglobin subunit alpha-1/2	HBA_RAT	8	411
Angiopoietin-like 6	B2RYM1_RAT	8	407
Alpha-2-HS-glycoprotein	FETUA_RAT	8	403
Uncharacterized protein	M0R9U2_RAT	6	403
Hemoglobin subunit beta-1	HBB1_RAT	6	380
Uncharacterized protein	A0A0G2K290_RAT	6	378
Uncharacterized protein	F1LZ11_RAT	7	373
Ig lambda-2 chain C region	LAC2_RAT	6	340
Uncharacterized protein	M0RAB8_RAT	5	330
Fetub protein	Q6IRS6_RAT	7	314
Uncharacterized protein	D4A3D1_RAT	5	312
Afamin	G3V9R9_RAT	7	312
Keratin, type I cytoskeletal 19	K1C19_RAT	7	308
Uncharacterized protein	A0A0G2JUY4_RAT	5	307
Uncharacterized protein	M0RDF2_RAT	5	281
Uncharacterized protein	M0RBK4_RAT	3	272
Uncharacterized protein	A0A0G2JY98_RAT	4	268
Uncharacterized protein	A0A0G2JXP0_RAT	4	257
Extracellular matrix protein 1	ECM1_RAT	5	255
Group specific component	Q68FY4_RAT	6	247
Desmoplakin	F1LMV6_RAT	6	244
Vitronectin	Q3KR94_RAT	6	233
Uncharacterized protein	M0RAV0_RAT	3	225
Peptidoglycan recognition protein 2	M0R485_RAT	5	220
Uncharacterized protein	A0A0G2K7I1_RAT	2	211
Uncharacterized protein	A0A0G2JW41_RAT	2	210
Uncharacterized protein	A0A0G2K7P6_RAT	3	204
Glutathione peroxidase	A0A0G2K531_RAT	4	201
Uncharacterized protein	A0A0G2JTG4_RAT	3	200
Uncharacterized protein	F1LVL4_RAT	3	199
C4b-binding protein alpha chain	C4BPA_RAT	6	196
RCG21066	D3ZJW6_RAT	4	194
Alpha-2-macroglobulin	A2MG_RAT	4	194
Uncharacterized protein	A0A0G2K304_RAT	4	193
Uncharacterized protein	A0A0G2K4I8_RAT	4	188
Uncharacterized protein	M0R693_RAT	4	187
Complement C1q subcomponent subunit B	G3V7N9_RAT	3	186
T-kininogen 1	KNT1_RAT	4	174
Uncharacterized protein	A0A0G2K332_RAT	3	169
Protein AMBP	AMBP_RAT	2	169
Uncharacterized protein	A0A0G2JZV7_RAT	3	164
Uncharacterized protein	A0A0G2JXB7_RAT	4	161
Uncharacterized protein	M0RD98_RAT	2	161
Junction plakoglobin	PLAK_RAT	2	154
Actin, cytoplasmic 1	A0A0G2K3K2_RAT	3	153
Uncharacterized protein	A0A0G2JV42_RAT	2	153
Uncharacterized protein	F1M7B3_RAT	2	151
Uncharacterized protein	A0A0G2K245_RAT	2	150
Uncharacterized protein	M0R4Z4_RAT	3	142
Uncharacterized protein	F1M1R0_RAT	2	138
Uncharacterized protein	F1LYF1_RAT	2	134
Complement C8 gamma chain	D3ZPI8_RAT	2	134
Uncharacterized protein	D3ZC54_RAT	2	133
Uncharacterized protein	F1M3E9_RAT	3	132
CD5 antigen-like	Q4KM75_RAT	2	129
Collagen type XVIII alpha 1 chain	F1LR02_RAT	2	128
Serpin family F member 2	F7FHF3_RAT	2	126
Uncharacterized protein	M0R628_RAT	2	124
Uncharacterized protein	G3V8Z5_RAT	3	118
Uncharacterized protein	F1LZH0_RAT	2	117
Ab1-233	A0A0H2UHI5_RAT	2	105
Papilin, proteoglycan-like sulfated glycoprotein	D3ZD40_RAT	3	104
Uncharacterized protein	A0A0G2K3K8_RAT	2	104
Uncharacterized protein	F1LWS4_RAT	3	103
Uncharacterized protein	D3ZPL2_RAT	1	101
Uncharacterized protein	D4A4L6_RAT	2	101
Uncharacterized protein	M0R816_RAT	1	99
Glutamine synthetase	GLNA_RAT	3	99
Uncharacterized protein	A0A0G2K5D2_RAT	2	98
Uncharacterized protein	F1M663_RAT	1	96
Uncharacterized protein	M0R8G6_RAT	1	93
Uncharacterized protein	M0R8Q4_RAT	1	91
Uncharacterized protein	F1LWD0_RAT	2	91
Similar to RIKEN cDNA	A0A0G2K896_RAT	2	86
Uncharacterized protein	M0R5A0_RAT	2	83
Uncharacterized protein	F1M5L5_RAT	2	77
Uncharacterized protein	D3ZFF8_RAT	2	75
Uncharacterized protein	A0A0G2K4K2_RAT	2	73
Prothrombin	G3V843_RAT	2	73
Uncharacterized protein	M0R7Q2_RAT	2	69
Uncharacterized protein	A0A096P6M7_RAT	1	65
Tripartite motif-containing 33	D3ZUK4_RAT	2	65
Uncharacterized protein	M0R8B5_RAT	1	61
Uncharacterized protein	M0R4C5_RAT	1	60
Ig kappa chain V region S211	KVX01_RAT	1	59
Uncharacterized protein	F1LTY5_RAT	1	57
Uncharacterized protein	M0R4G1_RAT	1	55
Suprabasin	F7FEM5_RAT	1	53
NADH:ubiquinone oxidoreductase subunit B10	D4A0T0_RAT	2	52
Complement C1q subcomponent subunit A	C1QA_RAT	1	52
Ciliary rootlet coiled-coil, rootletin	F1M6Q2_RAT	2	52
E3 ubiquitin-protein ligase HUWE1	A0A0G2JVW5_RAT	2	52
Complement factor properdin	B0BNN4_RAT	1	52
Uncharacterized protein	A0A0G2JX36_RAT	1	51
Tubulin beta-2B chain	TBB2B_RAT	1	51
Uncharacterized protein	A0A0G2K0N6_RAT	1	50
Cadherin-1	CADH1_RAT	1	46
Caspase 8-associated protein 2	D4A7V6_RAT	2	46
Phospholipase A2	A0A0G2JZ44_RAT	1	46
PEX5-related protein	F1LMT5_RAT	2	44
Collectin sub-family member 11	F1LSS7_RAT	1	44
Oxysterol-binding protein	A0A0G2K0D5_RAT	2	43
Uncharacterized protein	F1M6N0_RAT	1	42
Obscurin-like protein 1	OBSL1_RAT	2	42
Serum amyloid A protein	Q5M878_RAT	1	41
Proline-rich protein 5	A0A0H2UHJ6_RAT	1	41
Complement component C9	F7F389_RAT	1	39
Methyltransferase-like 3	Q4V8G6_RAT	1	36
Complement C8 alpha chain	D3ZWD6_RAT	1	35
Serine (Or cysteine) peptidase inhibitor, clade C (Antithrombin), member 1	Q5M7T5_RAT	1	33
Cell cycle checkpoint control protein RAD9B	RAD9B_RAT	1	33
Heparin cofactor 2	A0A0G2K8K3_RAT	1	33
Histone H4	H4_RAT	1	33
Uncharacterized protein (Fragment)	A0A096MK45_RAT	1	32
Phosphatidylinositol glycan anchor biosynthesis, class Q	Q642B8_RAT	1	32

^ⱡ^ Ions score is −10*Log(P), where P is the probability that the observed match is a random event. Individual ions scores > 31 indicated identity or extensive homology (*p* < 0.05). Protein scores were derived from ions scores as a non-probabilistic basis for ranking protein hits.

**Table 3:** Deiminated proteins in plasma-EVs of sham-treated rats (*Rattus norvegucus*), as identified by Figure 95. and LC-MS/MS analysis. Deiminated proteins in sham-treated rat plasma were isolated by immunoprecipitation using the pan-deimination F95 antibody. The resulting F95-enriched eluate was then analysed by LC-MS/MS and peak list files submitted to mascot. *Rattus norvegicus* species-specific peptide sequence hits are listed, showing number of sequences for protein hits and total score. Green highlighted rows indicate protein hits identified in sham-treated (control) rat plasma only.

Protein Name	Symbol	Sequences	Total Score (*p* < 0.05) ^ⱡ^
Serum albumin	A0A0G2JSH5_RAT	13	829
RCG36700	A0A0G2K3G0_RAT	13	654
Keratin, type II cytoskeletal 5	K2C5_RAT	14	624
Keratin, type I cytoskeletal 17	A0A0G2K9Q9_RAT	12	571
Keratin, type I cytoskeletal 14	K1C14_RAT	8	500
Actin, cytoplasmic 1	A0A0G2K3K2_RAT	8	461
Keratin, type II cytoskeletal 6A	K2C6A_RAT	8	401
Keratin, type I cytoskeletal 19	K1C19_RAT	9	391
Keratin 16	Q6IFU9_RAT	6	368
Heat shock protein HSP 90-beta	A0A0G2K793_RAT	9	337
Keratin, type II cytoskeletal 8	K2C8_RAT	6	330
Keratin, type II cytoskeletal 75	A0A0H2UHH5_RAT	7	320
Heat shock 70 kDa protein 1A	HS71A_RAT	5	312
Heat shock protein HSP 90-alpha	HS90A_RAT	7	298
Uncharacterized protein	A0A0G2K828_RAT	4	284
Ig gamma-2A chain C region	IGG2A_RAT	5	281
Isoform LMW of Kininogen-1	KNG1_RAT	6	272
Alpha-1-macroglobulin	A1M_RAT	8	267
Keratin, type I cytoskeletal 42	K1C42_RAT	5	265
Heat shock 70 kDa protein 1-like	HS71L_RAT	4	257
Complement C3	M0RBF1_RAT	6	238
Ig gamma-2C chain C region	IGG2C_RAT	5	224
Serotransferrin	A0A0G2QC06_RAT	5	222
Uncharacterized protein	D4ACR1_RAT	3	179
Murinoglobulin-1	A0A0G2JUP5_RAT	3	148
Ig kappa chain C region, A allele	KACA_RAT	2	142
Desmoplakin	F1LMV6_RAT	3	131
C-reactive protein	A0A0G2K8V5_RAT	2	119
Histone H4	H4_RAT	2	109
14-3-3 protein theta	1433T_RAT	2	103
40S ribosomal protein	RSSA_RAT	1	93
Elongation factor 1-alpha	M0R757_RAT	2	87
Ubiquitin-like modifier-activating enzyme 1	UBA1_RAT	2	87
Ig gamma-2B chain C region	IGG2B_RAT	2	82
Uncharacterized protein	A0A0G2JUY4_RAT	1	82
L-lactate dehydrogenase A chain	LDHA_RAT	2	69
Uncharacterized protein	M0RA79_RAT	1	66
Apolipoprotein E	A0A0G2K151_RAT	1	64
Globin a4	A0A0G2JSW3_RAT	1	63
Uncharacterized protein	A0A0G2K980_RAT	1	60
Uncharacterized protein	D3ZE63_RAT	1	57
Histone H2A.Z	H2AZ_RAT	2	55
Peptidyl-prolyl cis-trans isomerase FKBP4	FKBP4_RAT	1	55
Peroxiredoxin-2	A0A0G2JSH9_RAT	1	55
L-lactate dehydrogenase B chain	LDHB_RAT	2	54
Eukaryotic initiation factor 4A-II	A0A0G2K8B7_RAT	2	52
Clathrin heavy chain	F1M779_RAT	1	49
Tubulin alpha chain	A0A0H2UHM7_RAT	1	49
Uncharacterized protein	A0A096P6M7_RAT	1	46
cAMP responsive element binding protein 3, isoform CRA	A0A0G2K331_RAT	2	45
Transportin 1	F1LQP9_RAT	1	44
Unconventional myosin-Va	A0A0G2K4Y7_RAT	2	44
6-phosphogluconate dehydrogenase, decarboxylating	A0A0G2K7Q8_RAT	1	43
Uncharacterized protein	A0A0G2K8S2_RAT	1	41
Phospholipase A2	A0A0G2JZ44_RAT	1	41
Histone H2B	A0A0G2JXE0_RAT	1	40
Ventricular zone-expressed PH domain-containing protein homolog 1	A0A0G2K6S2_RAT	1	40
Uncharacterized protein	A0A0G2JVP4_RAT	1	37
Pyruvate kinase	A0A0G2JVG3_RAT	1	37
Centromere protein E	D3ZV60_RAT	1	36
Methyltransferase-like 3	Q4V8G6_RAT	1	35
Transketolase	G3V826_RAT	1	35
Uncharacterized protein	A0A0G2KB28_RAT	1	35
Enhancer of mRNA-decapping protein 4	EDC4_RAT	1	35
T-complex protein 1 subunit eta	D4AC23_RAT	1	34
Signal-induced proliferation-associated 1	E9PSX8_RAT	1	34
Stress-70 protein, mitochondrial	F1M953_RAT	1	32
Filamin-C	A0A0H2UHR7_RAT	1	32

^ⱡ^ Ions score is −10*Log(P), where P is the probability that the observed match is a random event. Individual ions scores > 31 indicated identity or extensive homology (*p* < 0.05). Protein scores were derived from ions scores as a non-probabilistic basis for ranking protein hits.

**Table 4:** Deiminated proteins in plasma-EVs of pre-motor PD rats (*Rattus norvegicus*), as identified by F95-enrichment and LC-MS/MS analysis. Deiminated proteins in pre-motor PD rat plasma were isolated by immunoprecipitation using the pan-deimination F95 antibody. The resulting F95-enriched eluate was then analysed by LC-MS/MS and peak list files submitted to mascot. *Rattus norvegicus* species-specific peptide sequence hits are listed, showing number of sequences for protein hits and total score. Orange highlighted rows indicate protein hits identified in plasma-EVs of pre-motor PD rats, but not in plasma-EVs of sham-treated rats.

Protein Name	Symbol	Sequences	Total Score (*p* < 0.05) ^ⱡ^
Serum albumin	ALBU_RAT	41	2238
Alpha-1-macroglobulin	A1M_RAT	33	1650
Keratin, type II cytoskeletal 5	K2C5_RAT	29	1395
Similar to histidine-rich glycoprotein	A0A0G2K9Y5_RAT	28	1305
Serotransferrin	TRFE_RAT	28	1238
Alpha-1-inhibitor	A1I3_RAT	21	976
Kininogen-1	KNG1_RAT	17	898
Complement C3	M0RBF1_RAT	16	815
Keratin, type II cytoskeletal 75	A0A0H2UHH5_RAT	13	714
Ig gamma-2C chain C region	IGG2C_RAT	14	699
Murinoglobulin-1	A0A0G2JUW7_RAT	15	683
Ig gamma-2A chain C region	IGG2A_RAT	12	670
Keratin, type I cytoskeletal 17	A0A0G2K9Q9_RAT	14	663
Keratin, type II cytoskeletal 6A	K2C6A_RAT	12	649
Keratin, type II cytoskeletal 1b	A0A0G2JZQ9_RAT	12	595
Keratin, type I cytoskeletal 14	K1C14_RAT	12	592
Actin, cytoplasmic 1	A0A0G2K3K2_RAT	11	539
Desmoplakin	F1LMV6_RAT	14	523
Keratin, type II cytoskeletal 8	K2C8_RAT	10	503
Junction plakoglobin	PLAK_RAT	10	481
Keratin, type I cytoskeletal 42	K1C42_RAT	11	472
Uncharacterized protein	A0A0G2JVP4_RAT	9	447
Uncharacterized protein	A0A0G2JVP4_RAT	7	417
Keratin 16	Q6IFU9_RAT	9	413
C-reactive protein	CRP_RAT	6	325
Uncharacterized protein	M0RA79_RAT	5	303
Tubulin alpha-1B chain	TBA1B_RAT	7	296
Uncharacterized protein	A0A0G2K9Y0_RAT	6	293
Ig kappa chain C region, A allele	KACA_RAT	6	280
Ac1873	Q7TQ70_RAT	7	256
Tubulin beta-5 chain	TBB5_RAT	5	246
Tubulin beta-2B chain	TBB2B_RAT	5	244
Tubulin beta-4B chain	TBB4B_RAT	5	229
Isoform Long of Annexin A2	ANXA2_RAT	3	228
Histone H4	H4_RAT	4	221
Uncharacterized protein	A0A0G2K290_RAT	4	218
Isoform Gamma-A of Fibrinogen gamma chain	FIBG_RAT	4	215
Carboxypeptidase N catalytic chain	CBPN_RAT	6	202
Uncharacterized protein	A0A0G2K828_RAT	3	176
Histone H2B	A0A0G2JXE0_RAT	3	172
Neurofilament heavy polypeptide	F1LRZ7_RAT	3	164
Keratin 83	A0A0G2JUU5_RAT	4	150
Uncharacterized protein	A0A0G2K3S3_RAT	2	137
Uncharacterized protein	M0RD98_RAT	2	132
Hemopexin	HEMO_RAT	4	130
Ig gamma-2B chain C region	IGG2B_RAT	3	129
Uncharacterized protein	D4ACR1_RAT	2	126
Carboxypeptidase N subunit 2	F1LQT4_RAT	3	123
Keratinocyte proline-rich protein	G3V9A5_RAT	3	122
Uncharacterized protein	F1LXY6_RAT	2	116
Plakophilin 1	D3ZY51_RAT	2	115
Selenoprotein P	SEPP1_RAT	3	106
Uncharacterized protein	F1M3X3_RAT	2	102
Apolipoprotein E	A0A0G2K151_RAT	3	97
Epsilon 1 globin	O88752_RAT	1	91
Globin c2	A0A0G2JSV6_RAT	2	82
Keratin 78 (Fragment)	A0A0G2JUR6_RAT	2	81
Isoform 2 of Fibrinogen beta chain	FIBB_RAT	2	81
Keratin, type I cytoskeletal 40	K1C40_RAT	2	80
Uncharacterized protein	A0A096P6M7_RAT	1	80
Neurofilament medium polypeptide	G3V7S2_RAT	2	73
Vitronectin	Q3KR94_RAT	2	72
Heat shock 70 kDa protein 1A	HS71A_RAT	2	72
Uncharacterized protein	A0A0G2K6T8_RAT	2	69
Uncharacterized protein	F1M6N0_RAT	2	66
Ras homolog family member C	A0A0G2K6E0_RAT	1	64
Chymotrypsinogen B	F1MA56_RAT	2	61
Uncharacterized protein	A0A0G2JZV7_RAT	1	59
Lactadherin	A0A0G2K506_RAT	1	57
Uncharacterized protein	A0A0G2JTG4_RAT	1	56
Uncharacterized protein	A0A0G2K099_RAT	1	51
Uncharacterized protein	A0A0G2JV42_RAT	1	51
Dystrophin	DMD_RAT	2	50
Cell division control protein 42 homolog	A0A0G2JSM8_RAT	1	50
Ig lambda-2 chain C region	LAC2_RAT	2	49
Uncharacterized protein	M0RBK4_RAT	1	43
Desmoglein 1	D3ZM39_RAT	1	42
Elongation factor 1-alpha	M0R757_RAT	1	42
Uncharacterized protein	A0A0G2K4K2_RAT	2	41
Cationic trypsinogen	G3V7Q8_RAT	1	41
Histidine ammonia-lyase	HUTH_RAT	1	40
Trypsin V-A	TRYA_RAT	1	39
Cingulin	D4A4X4_RAT	1	39
Uncharacterized protein	A0A0G2K8S2_RAT	1	39
Centrosomal protein 290	A0A0G2K715_RAT	1	39
Proline-rich protein 5	A0A0H2UHJ6_RAT	1	38
Uncharacterized protein	A0A0G2JUY3_RAT	1	38
Uncharacterized protein	M0RAB8_RAT	1	37
Spliceosome RNA helicase Ddx39b	A0A0G2KAT4_RAT	1	37
Uncharacterized protein	A0A0G2K0N8_RAT	1	37
Complement factor B (Fragment)	A0A096MKF9_RAT	1	36
Methyltransferase-like 3	Q4V8G6_RAT	1	35
Golgi apparatus protein 1	G3V8G5_RAT	1	35
Ceruloplasmin	A0A0G2K9I6_RAT	1	35
Histone H2A.Z	H2AZ_RAT	1	35
DNA-(apurinic or apyrimidinic site) lyase	D3ZPZ3_RAT	1	34
Keratin, type II cytoskeletal 80	K2C80_RAT	1	33
Uncharacterized protein (Fragment)	A0A096MK45_RAT	1	32

^ⱡ^ Ions score is −10*Log(P), where P is the probability that the observed match is a random event. Individual ions scores > 31 indicated identity or extensive homology (*p* < 0.05). Protein scores were derived from ions scores as a non-probabilistic basis for ranking protein hits.

## Supplementary Materials

The following are available online at https://www.mdpi.com/1422-0067/21/8/2743/s1, Table S1. LC-MS/MS analysis of F95-enriched proteins in plasma of sham-treated (control) rats. The detailed list of protein hits identified following protein immunoprecipitation using the pan-deimination F95 antibody in control rat plasma is presented. Table S2. LC-MS/MS analysis of F95-enriched proteins in plasma of pre-motor PD-model rats. The detailed list of protein hits identified following protein immunoprecipitation using the pan-deimination F95 antibody in control rat plasma is presented. Table S3. LC-MS/MS analysis of F95-enriched proteins in plasma-EVs of sham-treated (control) rats. The detailed list of protein hits identified following protein immunoprecipitation using the pan-deimination F95 antibody in control rat plasma is presented. Table S4. LC-MS/MS analysis of F95-enriched proteins in plasma-EVs of pre-motor PD-model rats. The detailed list of protein hits identified following protein immunoprecipitation using the pan-deimination F95 antibody in control rat plasma is presented. Figure S1. Deiminated histone H3 (citH3) is not detected in the substantia nigra of pre-motor PD rat brains. Immunostaining of substantia nigra is shown in sham and pre-motor rat brains indicating no positive immunodetection for citH3. A similar result was seen for F95 which also showed negative in SN both in sham and pre-motor PD brains (not shown); the scale-bar represents 100 µm.

## Abbreviations

AD	Alzheimer’s disease
ALS	Amyotrophic lateral sclerosis
BSA	Bovine serum albumin
CD63	CD63 antigen; granulophysin; lysosomal-associated membrane protein 3
ECL	Enhanced chemiluminescence
EVs	Extracellular vesicles
F95	Pan-deimination/citrullination antibody
FBS	Foetal bovine serum
Flot-1	Flotillin-1
HD	Huntington’s disease
KEGG	Kyoto encyclopedia of genes and genomes
LC-MS/MS	Liquid chromatography mass spectrometry
miR	microRNA
MS	Multiple sclerosis
NAFLD	Non-alcoholic fatty liver disease
NTA	Nanoparticle tracking analysis
PAD	Peptidylarginine deiminase
PD	Parkinson’s disease
SDS-PAGE	Sodium dodecyl sulfate polyacrylamide gel electrophoresis
SLE	Systemic lupus erythematosus
TBS	Tris buffered saline
TEM	Transmission electron microscopy
WB	Western blotting
