# Supplementary figures and images for: Protein Deimination Signatures in Plasma and Plasma-EVs and Protein Deimination in the Brain Vasculature in a Rat Model of Pre-Motor Parkinson’s Disease
Source: Int J Mol Sci. 2020 Apr 15;21(8):2743. doi: 10.3390/ijms21082743 (PMC7215947; doi:10.3390/ijms21082743)

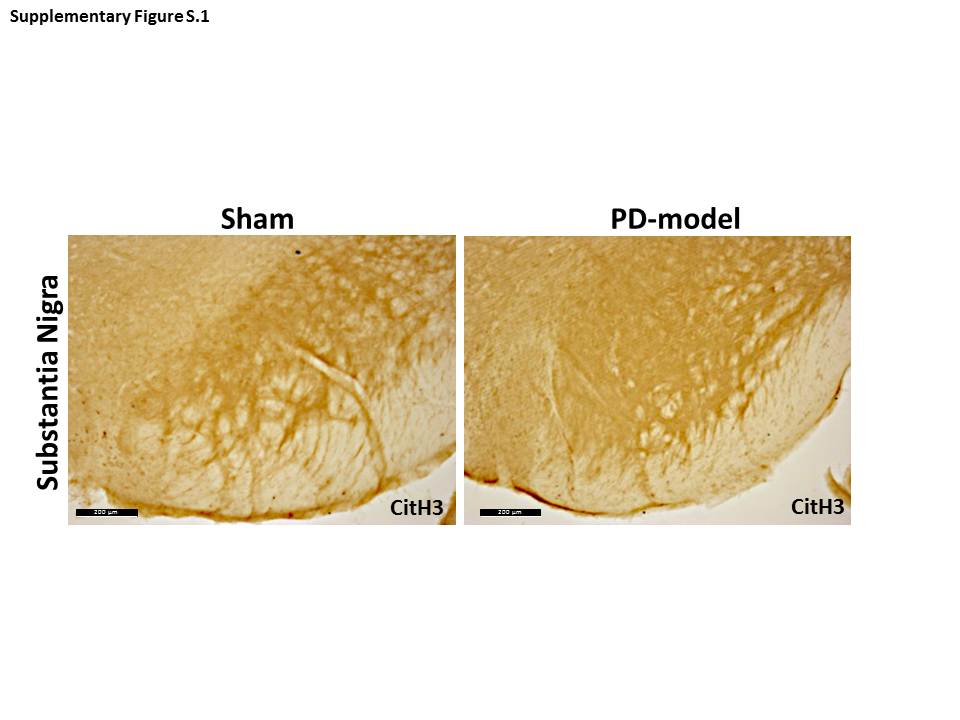

Supplement: Supplementary file 1 [file ijms-21-02743-s001.zip › Supplementary Figure S1.tif]
